# Effect of Removing Direct Payment for Health Care on Utilisation and Health Outcomes in Ghanaian Children: A Randomised Controlled Trial

**DOI:** 10.1371/journal.pmed.1000007

**Published:** 2009-01-06

**Authors:** Evelyn Korkor Ansah, Solomon Narh-Bana, Sabina Asiamah, Vivian Dzordzordzi, Kingsley Biantey, Kakra Dickson, John Owusu Gyapong, Kwadwo Ansah Koram, Brian M Greenwood, Anne Mills, Christopher J. M Whitty

**Affiliations:** 1 London School of Hygiene & Tropical Medicine, London, United Kingdom; 2 Dangme West District Health Directorate, Dodowa, Ghana; 3 Noguchi Memorial Institute for Medical Research, University of Ghana, Legon, Ghana; Harvard Medical School, United States of America

## Abstract

**Background:**

Delays in accessing care for malaria and other diseases can lead to disease progression, and user fees are a known barrier to accessing health care. Governments are introducing free health care to improve health outcomes. Free health care affects treatment seeking, and it is therefore assumed to lead to improved health outcomes, but there is no direct trial evidence of the impact of removing out-of-pocket payments on health outcomes in developing countries. This trial was designed to test the impact of free health care on health outcomes directly.

**Methods and Findings:**

2,194 households containing 2,592 Ghanaian children under 5 y old were randomised into a prepayment scheme allowing free primary care including drugs, or to a control group whose families paid user fees for health care (normal practice); 165 children whose families had previously paid to enrol in the prepayment scheme formed an observational arm. The primary outcome was moderate anaemia (haemoglobin [Hb] < 8 g/dl); major secondary outcomes were health care utilisation, severe anaemia, and mortality. At baseline the randomised groups were similar. Introducing free primary health care altered the health care seeking behaviour of households; those randomised to the intervention arm used formal health care more and nonformal care less than the control group. Introducing free primary health care did not lead to any measurable difference in any health outcome. The primary outcome of moderate anaemia was detected in 37 (3.1%) children in the control and 36 children (3.2%) in the intervention arm (adjusted odds ratio 1.05, 95% confidence interval 0.66–1.67). There were four deaths in the control and five in the intervention group. Mean Hb concentration, severe anaemia, parasite prevalence, and anthropometric measurements were similar in each group. Families who previously self-enrolled in the prepayment scheme were significantly less poor, had better health measures, and used services more frequently than those in the randomised group.

**Conclusions:**

In the study setting, removing out-of-pocket payments for health care had an impact on health care-seeking behaviour but not on the health outcomes measured.

Trial registration: ClinicalTrials.gov (#NCT00146692).

## Abstract 2

Evelyn Ansah and colleagues report on whether removing user fees has an impact on health care-seeking behavior and health outcomes in households with children in Ghana.

## Editors' Summary

**Background.:**

Every year, about 10 million children worldwide die before their fifth birthday. About half these deaths occur in developing countries in sub-Saharan Africa. Here, 166 children out of every 1,000 die before they are five. A handful of preventable diseases—acute respiratory infections, diarrhea, malaria, measles, and HIV/AIDS—are responsible for most of these deaths. For all these diseases, delays in accessing medical care contribute to the high death rate. In the case of malaria, for example, children are rarely taken to a clinic or hospital (formal health care) when they first develop symptoms, which include fever, chills, and anemia (lack of red blood cells). Instead, they are taken to traditional healers or given home remedies (informal health care). When they are finally taken to a clinic, it is often too late to save their lives. Many factors contribute to this delay in seeking formal health care. Sometimes, health care simply isn't available. In other instances, parents may worry about the quality of the service provided or may not seek formal health care because of their sociocultural beliefs. Finally, many parents cannot afford the travel costs and loss of earnings involved in taking their child to a clinic or the cost of the treatment itself.

**Why Was This Study Done?:**

The financial cost of seeking formal health care is often the major barrier to accessing health care in poor countries. Consequently, the governments of several developing countries have introduced free health care in an effort to improve their nation's health. Such initiatives have increased the use of formal health care in several African countries; the introduction of user fees in Ghana in the early 1980s had the opposite effect. It is generally assumed that an increase in formal health care utilization improves health—but is this true? In this study, the researchers investigate the effect of removing direct payment for health care on health service utilization and health outcomes in Ghanaian children in a randomized controlled trial (a trial in which participants are randomly assigned to an “intervention” group or “control” group and various predefined outcomes are measured).

**What Did the Researchers Do and Find?:**

The researchers enrolled nearly 2,600 children under the age of 5 y living in a poor region of Ghana. Half were assigned to the group in which a prepayment scheme (paid for by the trial) provided free primary and basic secondary health care—this was the intervention arm. The rest were assigned to the control group in which families paid for health care. The trial's main outcome was the percentage of children with moderate anemia at the end of the malaria transmission season, an indicator of the effect of the intervention on malaria-related illness. Other outcomes included health care utilization (calculated from household diaries), severe anemia, and death. The researchers report that the children in the intervention arm attended formal health care facilities slightly more often and informal health care providers slightly less often than those in the control arm. About 3% of the children in both groups had moderate anemia at the end of the malaria transmission season. In addition, similar numbers of deaths, cases of severe anemia, fever episodes, and known infections with the malaria parasite were recorded in both groups of children.

**What Do These Findings Mean?:**

These findings show that, in this setting, the removal of out-of-pocket payments for health care changed health care-seeking behavior but not health outcomes in children. This lack of a measured effect does not necessarily mean that the provision of free health care has no effect on children's health—it could be that the increase in health care utilization in the intervention arm compared to the control arm was too modest to produce a clear effect on health. Alternatively, in Ghana, the indirect costs of seeking health care may be more important than the direct cost of paying for treatment. Although the findings of this trial may not be generalizable to other countries, they nevertheless raise the possibility that providing free health care might not be the most cost-effective way of improving health in all developing countries. Importantly, they also suggest that changes in health care utilization should not be used in future trials as a proxy measure of improvements in health.

**Additional Information.:**

Please access these Web sites via the online version of this summary at http://dx.doi.org/10.1371/journal.pmed.1000007.

- This research article is further discussed in a *PLoS Medicine* Perspective by Valéry Ridde and Slim Haddad
- The World Health Organization provides information on child health and on global efforts to reduce child mortality, Millennium Development Goal 4; it also provides information about health in Ghana
- The United Nations Web site provides further information on all the Millennium Development Goals, which were agreed to by the nations of the world in 2000 with the aim of ending extreme poverty by 2015 (in several languages)
- The UK Department for International Development also provides information on the progress that is being made toward reducing child mortality

## Introduction

Levels of mortality in African children are unacceptably high. Access to medical care is a key determinant of health and one that can be addressed [1,2]. Malaria is a major contributor to childhood morbidity and mortality in children under 5 y of age [3]. In most settings where it has been investigated, the majority of children with symptoms compatible with malaria do not access formal health care [4,5]. Delay in seeking care can end in the death of a sick child before or shortly after they reach the clinic; almost all of these deaths should be avoidable if treated early [6]. More commonly, untreated or under-treated malaria can cause significant morbidity, especially anaemia. The same is true for many of the other major diseases of childhood.

Considerable efforts have been made to identify the barriers to accessing health care with the aim of increasing rapid access to health care in the public sector by those in need. Potential barriers include: perceived quality of service, socio-cultural factors, availability of health services, distance and travel cost, and cost of services [7–11]. Since access to early diagnosis and appropriate treatment of febrile illness is essential to preventing malaria morbidity and mortality, changes that influence the provision of prompt and effective treatment are of critical importance to malaria control efforts [4].

The financial cost of seeking health care has been shown in observational studies to be a major barrier to access to formal health care, especially among the poorest. Facilitating financial access to treatment is potentially one of the factors most amenable to intervention. This factor is likely to become increasingly important in sub-Saharan Africa due to drug resistance rendering cheap drugs ineffective; for example since the advent of drug resistant malaria, effective artemisinin-based antimalarials (ACTs) are generally only available to the poorest through public health outlets because of their high cost in the private sector. A review of Community Based Health Insurance (CBHI) in low-income countries found that CBHI schemes provide financial protection by increasing access to health care in the areas where they operate, but that no attempt had been made to assess the health benefits of introducing health insurance and that, the evidence base on CBHI had so far not included any randomized controlled trial study design [12]. Whilst community-based fees have some advantages, the negative effects of user fees on utilisation is well documented [13–19]. Removing user fees led to increased health care utilisation in South Africa and Uganda [20,21]. In Rwanda and Zaire, health facility data showed higher utilisation rates for the insured compared to the uninsured [13,22]. A study in the Volta Region of Ghana showed that, collection of user fees whilst enabling service provision to continue, ended up becoming a barrier to access to a significant number of poor patients. Fewer than one in 1,000 patient contacts resulted in exemptions being granted during the period of the study in 1995 [23]. The introduction of user fees in the early 1980s led to a drop in utilisation of health facilities in Ghana. Whilst with time, urban utilisation regained its prefee introduction level, utilisation in rural areas remained low [24].

Whilst there is a considerable literature of the effects of free care on health care utilisation, assessments of economic interventions have seldom looked at their impact on health outcomes [12]. A review of 82 health insurance schemes for people outside the formal sector found that none had been evaluated with regards to their impact on health [25]. Lagarde et al. found that although conditional cash transfers increased the use of health services, there were mixed effects on objectively measured health outcomes such as anaemia and some anthropometric measures [26].

There are good arguments based on equity to provide free health care, and it can be undertaken without undermining quality [21], but there is an opportunity cost to any decision and doing so has substantial and open-ended resource implications. Based on the observation that removing financial barriers increases formal health care utilisation, several countries are trying to implement free primary health care for all. There is an assumption by authors and policymakers that this model leads to better health outcomes, but this hypothesis is untested. Given the resources involved, determining the impact of free health care on health outcomes is currently a priority. We therefore aimed to determine by means of a randomised trial the impact of removing the direct cost of health care, including free provision of primary care, on health outcomes as well as health care utilisation in children. Malaria was chosen as the leading cause of morbidity and mortality in children under 5 y of age in Ghana [27,28]. This study set out to determine the impact of providing free primary health care on anaemia and other health outcomes, in children under 5 y of age in rural Ghana. Anaemia was chosen as the primary outcome because it is the most commonly used objective outcome of community interventions on malaria morbidity, with malaria the most common life-threatening disease of children under 5 y of age in West Africa [29–32].

## Methods

A household randomised, controlled unblinded trial of the impact of providing free primary health care, drugs, and initial secondary care on moderate anaemia in Ghanaian children under 5 y of age was undertaken. The study included a third observational arm of those who self-enrolled in a prepayment scheme.

### Study Area and Population

The study was carried out in the Dangme West District in southern Ghana, a rural district with an estimated 2004 mid-year population of 115,005 living in scattered communities of fewer than 2,000 people. There is widespread poverty in the area. The district has no hospital so inhabitants use five hospitals in surrounding districts for referral care.

Malaria treatment in the study area prior to the trial was presumptive treatment with chloroquine. In preparation for the trial, a standard World Health Organization (WHO) study of the efficacy of chloroquine was undertaken [33]. Only 42 (39.6%) of 106 children with malaria treated with chloroquine showed an adequate clinical and parasitological response to treatment. The district was, therefore, given permission to switch to amodiaquine + artesunate for malaria treatment in children prior to a national changeover to this drug combination for first line treatment of malaria, and this combination was used throughout the trial in both public and private-sector facilities.

Although in theory children under 5 y of age have been exempt from paying fees at public health facilities since 1997, a recent review showed that in practice only 6% of these children visiting public health facilities in Greater Accra Region, where the study was located, were exempted from paying fees for curative care [34]. In the study area the exemption policy was in practice not being implemented.

### The Intervention

The study intervention was provision of free health care to households randomised to the intervention arm by enrolling them into an existing prepayment scheme operating in the area [35], for which the study paid the fees. The Dangme West community prepayment scheme, a non-profit–making scheme, was designed jointly by the District Health Directorate, the District Assembly, and community members. It began operation in the year 2000 after a 4-y planning phase. It was initiated out of an identified need in the district of resident's inability to pay out-of-pocket fees, which affected their utilisation of health facilities. It covered the use of health services in the public sector. Membership was voluntary, with mandatory household registration (i.e., households were enrolled, not individuals). Members were allowed to use any of the ten primary care clinics whenever they fell ill and a referral hospital of their choice when referred. Every member of enrolled households received individual picture ID cards with a unique identification number. Enrolled members who fell ill were only required to present their ID cards at primary health care facilities in the district in order to receive free health services. For those who were in the control group, for a case of malaria that did not include a blood test the patient paid approximately 12,000 Ghana cedis (0.75 British pounds), whilst with a blood test included the patient paid around 17,000 Ghana cedis. The District Pharmacist monitored for drug stock-outs; none occurred in the study period.

Each household member in the intervention arm received a picture ID card, which allowed free access to primary care, including diagnosis and drugs with no limit, and more limited free access to secondary health care. The control arm paid user fees for their health care getting an equivalent benefit next year. Households who had voluntarily enrolled into the same prepayment scheme prior to the study provided a third observational arm [35]. Households were unable to change their group until the study ended in December of that year. Households belonging to the control arm were supported subsequently the following year together with those who enrolled of their own volition.

The trial was not announced until after the enrolment window for the scheme had closed and it was not possible for participants not randomised to free care to enter the scheme during the study period because enrolment can only occur once a year in this window period. Those who were going to self-enrol had therefore done so.

### Study End Points

The primary end point for the trial was the proportion of children with moderate anaemia (haemoglobin [Hb] < 8 g/dl) in the intervention and control arms detected during a cross-sectional survey at the end of the malaria transmission season. The main secondary outcome was the rate of utilisation of formal health care services by both intervention and control arms over the 6-mo period of the malaria transmission season. Additional secondary health outcomes were the prevalence of severe anaemia (Hb < 6 g/dl) and malaria parasitaemia at the end of the malaria transmission season, all-cause mortality, and anthropometric measurements.

### Sample Size

It was assumed, based on data collected previously in the study area, that the prevalence of moderate anaemia among the control group would be 10% at the end of the 24-wk period of follow-up. In order to detect an absolute difference of 4% in the prevalence of anaemia between the two groups, the sample size required to give a study with a power of 90% at a significance level of 5%, was a total of 2,028 children (1,014) in each arm. This sample size would also be able to detect a 0.3 g/dl difference in mean Hb concentration between the arms with similar statistical power. To allow for loss to follow up of 10% and the clustering effect of more than one eligible child in some households (mean 1.2, rho = 0.4), the aim was to recruit 2,500 children.

### Randomisation

All households with at least one child aged 6 to 59 mo who had not already enrolled in the prepayment scheme for the year were eligible to participate in the trial. The number of households with children under 5 y of age in the study area was about 8,700; 2,332 of these households were randomly selected for inclusion in the study using computer-generated random numbers from the district health directorate database. All randomly selected households were visited and households were excluded from the trial if there were no children <6 y of age in the household, parental consent was refused, or households were due to emigrate from the study area within the coming 2 y. Households that had previously paid to enrol in the prepayment scheme were excluded from the trial but, if they consented, were enrolled as an observational comparator arm.

A stratified randomisation procedure was used [36]. Households were divided into three strata based on distance of residence from a health facility being ≤5 km, 5–10 km, and >10 km, respectively, since distance from a health facility is known to be a major determinant of its use. The aim was to reduce the potential confounding that distance would introduce.

At the meeting all heads of households or their representatives were allocated serial numbers. An equal number of folded papers with “Yes” or “No” written on them totalling the number present were dropped into a rotating barrel and mixed up thoroughly in the view of all. Each household head was then invited to pick the papers by calling out their numbers. Those who picked “Yes” were assigned to the intervention group and those who picked “No” to control. This process was used to make the trial more acceptable to community members by showing them the lack of favouritism and randomness of the allocation*.* Households could not change their group until the study ended in December.

Households in the control arm were enrolled in the insurance scheme in the year following the trial for a period of one year as were those who had previously paid to enrol in the health insurance scheme. Enrolment was arranged directly with the Health Insurance Scheme and households were required to make themselves available for their pictures to be taken after which they received their cards with which they could access health care.

### Survey Methods

Questionnaires were translated into the local language and back-translated. Pictorial diary and other data collection tools were pretested on two occasions in two areas outside the area where the study was to take place. Wealth strata were constructed by determining an asset index by direct observation in the home and determining wealth quintiles using principal component analysis [37,38].

Utilisation of different health services was assessed by documented household pictorial diaries supplied to households and collected by fieldworkers on a monthly basis during a 6-mo period of follow-up. These data were used to assess the person-years of follow-up. During cross-sectional surveys, children were weighed naked using an infant weighing scale. The length of children <24 mo old was recorded using an infantometer; a stadiometer was used for children >24 mo of age. The MUAC (mid-upper-arm circumference) of each child was measured using Shakir's strip. Measurements were carried out twice and the mean used for analysis.

### Laboratory Methods

Finger-prick blood samples were obtained for measurement of Hb concentration using a Haemocue haemoglobinometer and microcuvettes (Haemocue AB)*.* Thick and thin blood films for malaria were stained with Giemsa and examined by two microscopists blind to the study group to which the slide belonged with a third read for discrepant slides. The third reading was the definitive one if close to any of the earlier two read. 100 fields were examined before a film was considered negative. Children with fever or reported fever in the past week were tested immediately for malaria by means of a dipstick antigen capture test*.* Children found during the baseline cross-sectional survey to have a Hb concentration less than 8 g/dl, fever, or a history of fever and parasitaemia were treated according to local guidelines but retained in the study.

Children found to have anaemia during the final cross-sectional survey were investigated further to determine alternative, nonmalarial causes of anaemia. Hb electrophoresis, glucose-6-phosphate dehydrogenase (G6PD) testing, full blood count, concentrated stool and urine microscopy were undertaken. Agarose gel electrophoresis of an eluate from a filter paper blood spot was used to determine Hb genotype. For the G6PD test, 1 ml of whole blood was put in a test tube together with 50 μl each of methylene blue and GPPD solution. The tube containing the test was compared with positive and negative standard tubes. To assess whether study participants had been taking chloroquine during the previous 3–4 mo, chloroquine was assayed in urine using a validated dipstick [39] among a random selection of 925 participants during the final cross-sectional survey. The participants were selected by means of computer generated random numbers. 400 were randomly selected from the intervention and control arms whilst the rest were selected from the 125 households who had voluntarily enrolled in the health insurance scheme. As a check of ongoing efficacy of amodiaquine + artesunate treatment all patents with a fever reported in the last week had a rapid diagnostic test performed, and were treated by a study nurse; at day 14 following treatment all were found to be slide-negative.

### Data Management and Statistical Methods

Data were entered into EpiInfo6 and analysed using STATA9. Summary statistics, odds ratios (ORs), confidence limits, and *p*-values were calculated to compare outcomes between the two randomized groups for the primary and secondary endpoints. A major secondary analysis was performed comparing those who had enrolled and paid voluntarily with the two other groups. Stratified analyses were carried out on the basis of distance of household residence from a health facility and household wealth. ORs for the primary and secondary outcomes were calculated unadjusted and adjusted for predefined potential confounding factors in a logistic regression model, and clustering by household was adjusted for using a population-averaged Generalized Estimating Equation (GEE) model for both unadjusted and adjusted primary outcome. The predefined potential confounding factors were age, sex, distance of household residence from a health facility, and socioeconomic status defined by wealth quintile. An analysis was undertaken to assess whether children for whom there were missing data differed in terms of anaemia and other key indicators from children who completed follow-up. To allow for clustering within households, the other main analyses were repeated using the svy command in STATA setting households as the primary sampling unit (psu). For the primary outcome *p* < 0.05 (two-sided test) was taken to be significant.

### Qualitative Data

Perceptions of quality of primary health care among study participants were investigated by focus-group discussions in the community, and exit interviews of patients/parents attending primary health care facilities conducted by field-workers not involved in clinical care. Detailed description of methods and results of additional qualitative data will be presented elsewhere, but are available from the authors on request.

### Ethical Review, Sponsorship, and Protocol

Ethical approval was obtained from the Ethical Review Committees of the Ghana Health Service and the London School of Hygiene & Tropical Medicine. A CONSORT statement (Text S1) and the initial protocol (Text S2) are available as additional information.

## Results

**Figure 1:**
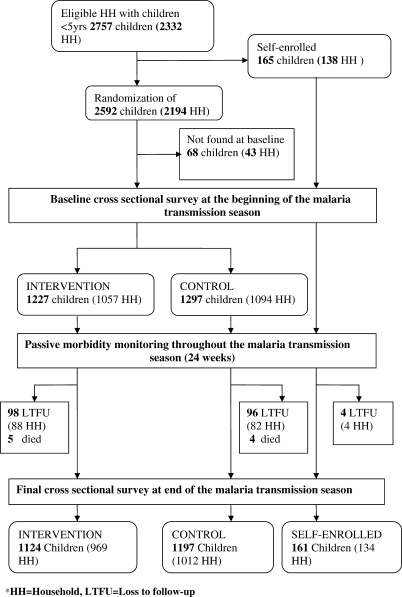
Trial Profile

**Figure 2:**
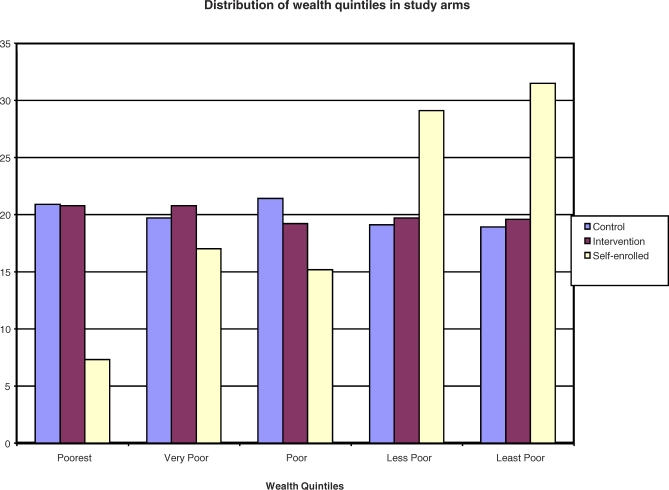
Distribution of Wealth Quintiles in Study Arms

The study ran from May 2004 to February 2005. 2,332 households in Dodowa and Prampram subdistricts with 2,757 children aged 6–59 mo randomly selected from a district database to participate in the trial. No household refused consent. 138 of these households with 165 children had already enrolled voluntarily in the prepayment scheme at the time of closure of the registration window; all agreed to take part in the observational arm. The remainder, 2,194 households with 2,592 children were randomised and included in the trial. A total of 2,524 children from 2,151 households participated in the baseline cross-sectional survey, 1,227 from the intervention and 1,297 from the control arm. 68 children were unavailable due to travel. Follow-up at the final cross sectional study was 92% in the intervention and 93% in the control arms, respectively (Figure 1). Households in the intervention and control arms were similar at baseline (Table 1). However, the self-enrolled group were different from the randomised groups both in socioeconomic and health status at the start of the trial (Table 1). The trial groups were evenly distributed across the wealth quintiles, but the self-enrolled group was skewed toward the wealthier quintiles (Figure 2).

**Table 1:**
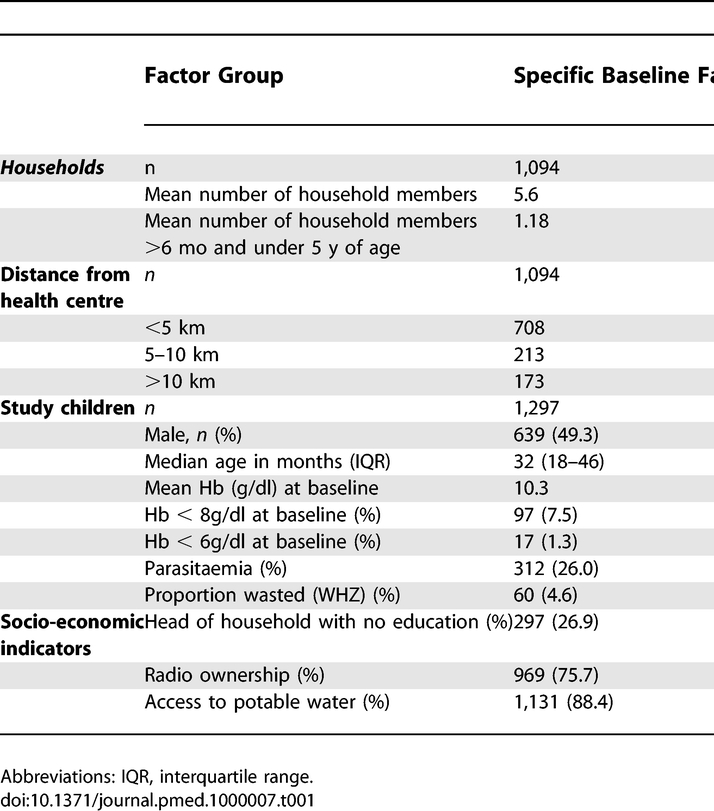
Comparisons at Baseline of Control, Intervention, and Self-Enrolled Study Arms

An exit interview on quality of care found 147/161 (91%) indicating they perceived excellent or good service, compared to five (3%) who indicated a poor service. Results of focus group discussions of quality of care among mothers of children in the study in the community (whether they had attended clinic or not) also indicated a high level of satisfaction with the quality of care provided by primary care facilities in the district. These results will be presented in more detail elsewhere. An analysis assessing whether children for whom there were missing data differed in terms of anaemia and other key indicators from children who completed follow-up showed no differences between these two groups.

### Impact on Health Care Utilisation for Curative Care

As anticipated, utilisation of formal primary health care dropped, and use of informal health care increased with distance from a health centre in both intervention and control arms (Table 2). Informal care refers to any health care other than that from clinic, health centre, or hospital, and includes traditional healers, chemical sellers, and home remedies. Similarly, there was a gradient in utilisation of both formal and informal health care by wealth quintile with the poorest more likely to use informal care in both intervention arms.

**Table 2:**
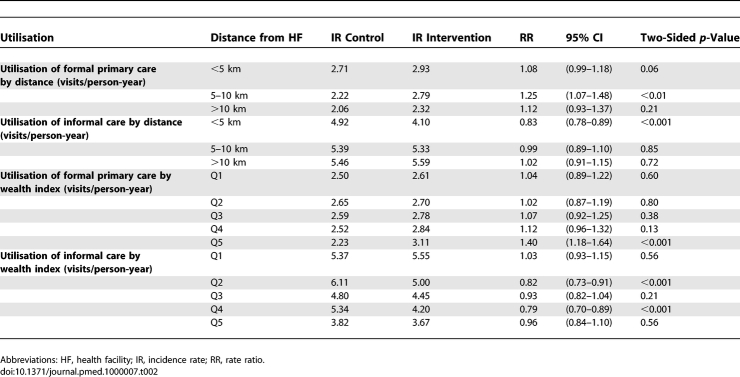
A Comparison of the Utilisation of Primary and Nonformal Care Services between Households in Intervention and Control Arms by Distance of Residence from the Nearest Health Facility and Wealth Quartile

Providing free health care had a modest, but significant, impact on health care utilisation (Table 3). Children were taken to primary care facilities significantly more frequently in the intervention arm (2.8 episodes per person-year) than in the control arm (2.5 episodes per person-year), rate ratio 1.12 (95% confidence interval [CI] 1.04–1.20; *p* = 0.001). Families with free health care sought care for illness from a chemical seller and treated children at home significantly less frequently than those who had to pay at the primary health care facility. Children in the intervention arm utilized nonformal services as a whole significantly less frequently (4.59 per person-year) than those in the control arm (5.1 per person-year), rate ratio 0.90 (95% CI 0.86–0.95; *p* < 0.001).

**Table 3:**
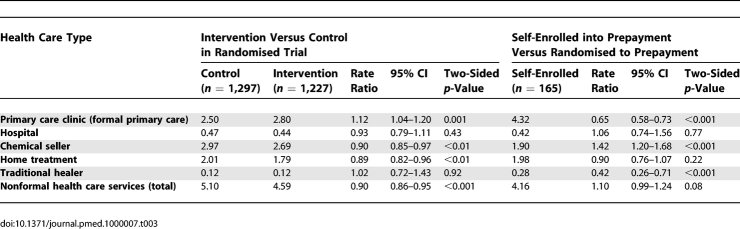
Comparison of the Utilisation of Health Care by Households in the Control Compared to the Intervention, and the Self-Enrolled into the Prepayment Scheme Compared to Those Randomised to the Prepayment Scheme

A comparison of those provided with free treatment through the intervention and those who self-enrolled into the insurance scheme before the study (who had identical access to free health care) showed that households in the intervention group utilized primary care services substantially less than the 4.3 visits/person-year recorded among the self-enrolled group, rate ratio 0.65 (95% CI 0.58–0.73; *p* < 0.001). The self-enrolled used chemical sellers significantly less, but surprisingly traditional healers more than those in the intervention arm (Table 2).

### Impact on Health Outcomes

There was no significant difference in the mean number of fever episodes per person-year between children in each of the three study arms (5.72, 5.53, and 5.83 in the control, intervention, and self-enrolled arms, respectively). There were nine deaths among study children during the 6-mo follow-up period of the study, four in the control arm, five in the intervention arm, and none in the self-enrolled arm.

At the end of the malaria transmission season cross-sectional survey, there were no differences between intervention and control children in the prevalences of moderate anaemia (Hb < 8 g/dl), severe anaemia (Hb < 6 g/dl), parasitaemia, or in mean Hb concentrations (Table 4). Thirty-six (3.2%) children in the intervention arm were anaemic at the end of the transmission season compared to 37 (3.1%) in the control arm, OR 1.04 (95% CI 0.66–1.66, *p* = 0.86), using GEE model taking account of clustering. Adjustment for age, sex, distance, and wealth quintile had no effect on outcome (OR 1.05, 95% CI 0.66–1.67). The mean Hb concentration of children in the intervention arm was 11.1 g/dl and in the control arm was 11.0 g/dl (*p* = 0.45 using GEE model based on household). Overall, there was a modest increase in mean Hb concentration in all three study arms at the end of the peak malaria transmission season compared with concentrations at the end of the dry season. The mean change in Hb concentration among children who belonged to the intervention arm was slightly higher (+0.75 g/dl) than in the control arm (+0.71 g/dl), but this difference does not achieve statistical significance (*p* = 0.69). 71 of 73 children found to be anaemic at the final survey were screened for alternative causes of their anaemia; ten had hookworm infection, 18 ascaris, and one had phenotypic glucose-6-phosphate dehydrogenase (G6PD) deficiency. Hb electrophoresis was undertaken in all the 71 children; 51 were AA, six AS, four AC, one CC, seven SS, and two SC. Anaemia is often multifactorial, and the finding of other potential causes does not exclude malaria contributing.

**Table 4:**
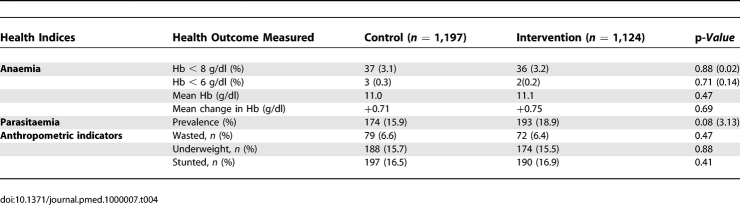
Effect of the Intervention on Health Indices Measured at the Final Cross-Sectional Survey

The prevalence of malaria asexual parasitaemia at the post malaria transmission season was similar in each group (Table 4), 101 children with fever or histories of fever had a positive rapid diagnostic test for malaria and were treated with amodiaquine and artesunate. All 86 who were traced 14 d later had cleared their parasitaemia. Only four of the urine samples of the 924 study participants gave a positive result with a dipstick antigen assay for chloroquine indicating that this drug was being used very little in the study area by study end. Anthropometric measurements were similar in children in each study arm (Table 4).

There was an overall increase in the outpatient attendance by children under 5 y old during the period of the study, with the number of cases seen in 2003, 2004, 2005, and 2006, 12,226, 24,058, 13,239, and 13,241 respectively. Adult attendance remained stable over this period.

## Discussion

This trial in rural Ghana found that children in households randomised to free healthcare used formal healthcare more and informal healthcare less than a control group. This utilisation did not translate into any change in anaemia (the primary outcome), mortality, or other health outcomes measured. An observational group who had paid to self-enrol into the same scheme were wealthier, healthier, and used both formal and informal healthcare more than those randomised to it at baseline and subsequently. A number of studies in both developed and developing countries have investigated the impact of lowering direct financial barriers to health care on utilisation, but this is the first randomised trial to investigate the impact of providing free health care on health outcomes. It used malaria-associated health outcomes in children as the indicator of health impact as it is the most important cause of serious childhood mortality and morbidity in the area. The failure to find any demonstrable health benefit from the change in utilisation following free health care was demonstrated even for those living within 5 km of a health care facility (so with limited physical barriers to access). This lack of any effect, including on secondary outcomes such as Hb for which the study had good power, challenges the assumption that where introducing free health care leads to changes in utilisation, it can safely be assumed to translate into health benefits. Given the potential size of resources involved in providing free health care that could be diverted from other priorities on the basis of that assumption, this finding is potentially important for policymakers.

This lack of any effect of being randomised to free health care on health outcomes is unexpected, since there has always been an assumption that increased access as a result of free health care improves health. The investigators have considered a number of reasons why the lack of effect may have occurred. It is possible that user fees may not be the major financial barrier to care in the formal health sector in the study area, so removing them may have had relatively limited impact. Indirect costs, including opportunity costs (e.g., the cost to the household of time spent away from work), may be more important and are not so easily modifiable as direct costs of care [8,40]. If some patients cannot afford to access the free care because of indirect costs, this is likely to have had its greatest effect on the poorest, who are also those who stand to gain most from effective medical care. Studies in other parts of Ghana and elsewhere have estimated that indirect costs are 2–3.6 times higher than the direct costs of the total cost of seeking treatment, making up on average 79% of the total cost of treatment [41–44,]. Variables such as distance from the health care facility [17,45], lack of knowledge, or incorrect perception of health care services and when to utilise them may also be important in determining health-seeking behaviour and will not be affected by removing user fees [46,47]. The fact that users reported high levels of satisfaction with the care they received, and that these ratings were confirmed by focus group discussions, make it unlikely that perceived poor health care provided by the health centres frequented by the study participants was the main reason for a lack of effect on health outcomes.

Anaemia was used as the primary health indicator, which, in the study area, is due in large measure to malaria. The failure of the intervention to show an impact on anaemia was not due to inadequate treatment of malaria at health facilities as ACT treatment was introduced prior to the start of the trial and shown during the course of the trial to be effective.

It is possible that the introduction of this effective antimalarial treatment in the study area just prior to the trial and ongoing vector control may have reduced transmission and resulted in a better health outcome for malaria in all study participants, but this result would have been expected to dilute rather than remove the effect completely. The new treatment cost more to end users in the paying arm than chloroquine, so the relative benefits of the free health care arm were greater during the trial than previously. However, in spite of the slightly increased treatment cost, there was a general perception of better efficacy. There was a slight increase of 5.2%, 4.2%, and 7.2% in insecticide-treated net use among the control, intervention, and self-enrolled arms, respectively over the study period, but these increases were not significantly different. No deworming programmes (another factor that could have influenced the prevalence of anaemia) occurred during the period of the study. The introduction of a very effective antimalarial, perceived to be so by the communities, may have resulted in the reduction in the disparity between utilisation in the two groups. The difference in health care utilisation was small, even though statistically significant, and this may have reduced the effect expected from the intervention. Although we did not demonstrate that provision of free health care had a significant positive impact on the health indicators that we measured, it is possible that there may have been improvements in other indicators that we did not measure. No trial of an intervention can be assumed to be generalisable to other settings, and it is possible that in other settings a positive outcome would have been seen.

Those individuals who had chosen to buy their own (identical) health insurance started the study period healthier than those who were randomised to have it provided free, and they used health care substantially more throughout the study period. Some of the literature supporting health outcomes being associated with free care comes from observational studies of individuals enrolled in insurance schemes, but these studies may be misleading; those who self-enrolled in a prepayment scheme were systematically different than those who did not and had identical benefits to the prepayment scheme provided free.

**Figure 3:**
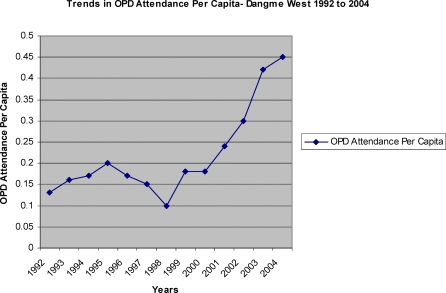
Trends in OPD Attendance Per Capita in Dangme West, 1992–2004

Any study that collects data in an operational trial runs the risk of contaminating the result because the fact of observation may change behaviour (the Hawthorne effect). In this study it is possible that the fact that data were being collected could have changed utilisation, although it will not have changed the outcome measures (anaemia, Hb, mortality), which are objective. It is therefore unlikely to explain the fact that an increase in utilisation led to no change in health care outcomes. There is also a theoretical reduction of effect from a time-lag between an intervention being introduced and its having an effect, and this lag could have had an impact on mortality (a secondary outcome); however it is unlikely to have extended to the primary outcome as Hb was measured after more than 6 mo of free health care. Utilisation rates among the control group did not differ from the trend seen in previous years in Dangwe West (Figure 3). It is possible the intervention would have shown an effect in another setting. The study demonstrates that economic evaluations should investigate impact on health outcomes directly rather than assuming utilisation is a safe proxy for increased health; it does not show that free health care in general will not have an impact on health in any setting.

Whilst there are good reasons based on equity for considering it, providing free health care to all is a substantial and open-ended commitment, so evidence of effectiveness needs to be demonstrated rather than assumed. Other changes such as improvements of the standard of care provided and the availability of transportation may need to occur in parallel if free health care is to have an effect. Providing free health care should not be considered an easy fix for the undoubted inequities in access to care. Direct cost of care is a barrier to the poorest in accessing care, but it is not the only one, and other modifiable barriers have to be addressed if removing the direct cost of care is to have a useful impact on the health of the poorest.

## Supporting Information

Text S1 CONSORT Statement (61 KB DOC)

Text S2 Original Protocol (332 KB PDF)

## Abbreviations

ACT: artemisinin-based antimalarial
CBHI: Community Based Health Insurance
CI: confidence interval
GEE: Generalized Estimating Equation
Hb: haemoglobin
OR: odds ratio
