# Correction: Effect of Removing Direct Payment for Health Care on Utilisation and Health Outcomes in Ghanaian Children: A Randomised Controlled Trial

**DOI:** 10.1371/journal.pmed.1000033

**Published:** 2009-02-24

**Authors:** Evelyn Korkor Ansah, Solomon Narh-Bana, Sabina Asiamah, Vivian Dzordzordzi, Kingsley Biantey, Kakra Dickson, John Owusu Gyapong, Kwadwo Ansah Koram, Brian M Greenwood, Anne Mills, Christopher J. M Whitty

Correction for:

Ansah EK, Narh-Bana S, Asiamah S, Dzordzordzi V, Biantey K, et al. (2009) Effect of removing direct payment for health care on utilisation and health outcomes in Ghanaian children: A randomised controlled trial. PLoS Med 6(1): e1000007. doi:10.1371/journal.pmed.1000007

In the author byline of the published article, the institutional affiliation of John Owusu Gyapong, seventh of the listed authors of the manuscript, was incorrectly indicated to be the London School of Hygiene & Tropical Medicine. John Owusu Gyapong is in fact based at the Health Research Unit of the Ghana Health Service in Accra, Ghana.

