# Supplementary material for: Effect of Removing Direct Payment for Health Care on Utilisation and Health Outcomes in Ghanaian Children: A Randomised Controlled Trial
Source: PLoS Med. 2009 Jan 6;6(1):e1000007. doi: 10.1371/journal.pmed.1000007 (PMC2613422; doi:10.1371/journal.pmed.1000007)
Supplement: Supplementary file 1 [file pmed.1000007.sd002.pdf]

LONDON SCHOOL OF HYGIENE AND TROPICAL MEDICINE
(UNIVERSITY OF LONDON)

1899 1999

contributing to health worldwide

A Randomized Controlled Trial Of The Impact Of Improved Financial Access To Health Care On Morbidity Due To Severe Malaria And Healthcare Utilization Among Children 6 Months To Five Years Of Age In A Malaria Endemic Area In Ghana

---

Principle Investigator **Evelyn Ansah MD, MPH**

AUGUST 5, 2004

An open randomized controlled trial to measure the impact of improved financial access to health care on malaria control among children 6 to 59 months in Sub-Saharan Africa.

Principal Investigator

Dr. Evelyn Ansah[1 2]

Co-investigators

Dr. Christopher Whitty[1]
Dr. Kwadwo Koram [4]
Dr. John Gyapong[3]
Prof. Anne Mills[1]
Prof. Brian Greenwood[1]

Affiliations

1. London School of Hygiene and Tropical Medicine
2. Dangme West District Health Administration/Research Centre
3. National Health Research Unit, Ministry of Health, Ghana
4. Noguchi Memorial Institute for Medical Research

Sponsor

Gates Malaria Partnership

Clinical Monitor

Dr. Onike Rodrigues, Head of Dept of Child Health, Korle Bu Teaching Hospital

Trial Duration

April 2003 to June 2005

STUDY SYNOPSIS

AIM

To improve malaria control in children less five years of age. The study intends to examine how improvement in financial access to health care can lead to a reduction in morbidity due to severe malaria among children six to fifty-nine months in a malaria endemic area.

OBJECTIVES

To assess by means of an open randomized trial, the impact of improving financial access to health care on utilization of health services and morbidity due to severe malaria among children six to fifty-nine months of age.

1.3.1 Primary objective

To compare the prevalence of anaemia (Hb<8g/dl) among children six to fifty-nine months of age, from households randomized to improved financial access or without for one year.

1.3.2 Secondary objectives

1. To compare the proportions of children six to fifty-nine months of age with severe anaemia (Hb<6 g/dl) from households with or without improved financial access.
2. To compare the health service utilization rate among children six to fifty-nine months of age from households with or without improved financial access.
3. To compare the mean haemoglobin concentration among children six months to fifty-nine months of age from households with or without improved financial access.
4. To compare anthropometric indicators among children six to fifty-nine months of age from households with or without improved financial access.
5. To compare child (6 to 59 months) as well as household all cause mortality among households with or without improved financial access.
6. To examine primary and major secondary outcomes by socio-economic status of households.
7. To compare primary and major secondary outcomes among children from self-enrolled and trial-enrolled households.
8. To document community knowledge, attitude and practice with regards to severe malaria, user fees, exemptions, the existing pre-payment scheme and indicators of poverty.

THE INTERVENTION

There will be two arms. The intervention group will be enrolled into a pre-payment scheme operating in the area which will allow them free access to primary care including diagnosis and drugs whenever they are ill with no limit, and limited access to secondary health care.

The control group will have to pay user fees for their health care as pertains normally. They will receive equivalent support the following year.

BACKGROUND DESCRIPTIVE STUDIES

An initial qualitative study will be carried out to provide background information on community perceptions of malaria and financing of health care for the study and set it in context.

OTHER PRELIMINARY STUDIES

An assessment of the therapeutic efficacy of chloroquine will be carried out in order to ascertain the efficacy of chloroquine in the study area. Chloroquine remains the first line antimalarial drug in the area but is in the process of being changed.

THE INTERVENTION TRIAL

A baseline cross-sectional survey will be carried out at the beginning of the peak malaria transmission season followed by passive morbidity monitoring at household, primary care clinics and referral hospitals level for the intervening 24 weeks. A final cross-sectional survey will be carried out in November at the end of the peak malaria transmission season.

Data will be collected during the trial from eligible households who voluntarily enrolled in the pre-payment scheme in order to document the differences between them and the randomized group.

SAMPLE SIZE

The required sample size is a total of 2028 children between 6 to 59 months of age (1014 in each arm)

STUDY SITE

The Dangme West District, rural Ghana

ETHICAL CONSIDERATIONS

Ethical approval will be obtained from the Ethics committees of both the Ghana Health Service (GHS) in Ghana and the London School of Hygiene and Tropical Medicine (LSHTM). Informed

consent will be sought from parents or guardians of all study participants before their inclusion in the study.

CURRENT STATUS OF STUDY

The assessment of therapeutic efficacy of chloroquine has been completed. This was followed by background descriptive studies to document community knowledge, attitude and practice with regards to severe malaria, user fees, exemptions, pre-payment schemes and indicators of poverty. Randomization of households by community ballot has been carried out after which a baseline cross-sectional survey collecting data on household socio-economic status as well as anthropometric and haematological data on children 6 to 59 months in the selected households has been completed. Passive morbidity monitoring of illness episodes of the children at household, primary care and referral hospital level is currently on-going. Ethical approval was obtained for the study from the Ethics committees of both the LSHTM and the GHS, local authorities and the parents/guardians of the children.

TABLE OF CONTENTS

TABLE OF CONTENTS 6
1.0 BACKGROUND TO THE STUDY 7
1.1 The burden of malaria in Ghana 7
1.2 Improving access to effective treatment for malaria 7
1.3 Methods of overcoming financial barriers to obtaining effective treatment 10
2.0 RATIONALE OF THE STUDY 13
3.0 OBJECTIVES 13
4.0 METHODS 15
4.1 Study Design 15
4.2 Study Site 15
4.3 Study Population 17
4.4 Inclusion and exclusion criteria 18
4.5 Sample size calculation 18
4.6 Screening and enrolment procedure 18
4.7 Randomization and allocation of intervention 18
4.8 Initial cross-sectional survey 19
4.9 Passive surveillance for malaria 20
4.10 Final cross-sectional survey 21
4.11 Preliminary Assessment of Therapeutic Efficacy of Chloroquine 21
4.12 Background descriptive studies 21
4.13 Measurement of study end points 22
4.14 Training for field work 22
5.0 DATA MANAGEMENT AND ANALYSIS 23
6.0 ETHICAL ISSUES 24
6.1 Ethical Approval 24
6.2 Ethical Considerations 24
6.3 Informed Consent 25
6.4 Confidentiality 25
7.0 ADMINISTRATIVE MATTERS 25
7.1 Responsibilities of the Principal Investigator 25
7.2 Protocol Amendments 26
8.0 TIME LINES AND MILESTONES FOR ACHIEVING OBJECTIVES 27
9.0 PRELIMINARY RESULTS .... **Error! Bookmark not defined.**
10.0 FUTURE WORK .... **Error! Bookmark not defined.**
11.0 CONCLUSIONS .... **Error! Bookmark not defined.**
12.0 REFERENCES 28
13.0 APPENDICES 31

1.0 BACKGROUND TO THE STUDY

1.1 The burden of malaria in Ghana

Malaria causes 140-280 million clinical cases and one million deaths each year among children under five. Severe malaria in children presents mainly as severe anaemia or cerebral malaria [1,2]. In Ghana, malaria is the most commonly reported disease at outpatient departments (OPD) and accounts for approximately 40% of total OPD attendance and 25% of deaths among children less than five years of age [3]. It is also the single most important contributor to the greatest number of "healthy days of life lost" in Ghana [4].

1.2 Anaemia as a measure of malaria morbidity

The WHO defines severe malarial anaemia as a haemoglobin count of less than 6 g/dl or a packed cell volume less than 15% in the presence of *Plasmodium falciparum* infection [5]. It is estimated that there are 1.42 to 5.66 million cases of severe malaria each year with a greater than 13% case fatality among children under 5 years of age [6].

Though anaemia is a multifactorial problem, and may be as a result of several factors including dietary deficiencies, helminths and other infections and several factors may operate in the same individual[7,8]. Sickle cell disease has also been recognized as an important risk factor for anaemia in sub-Saharan countries[8,9]. Studies have shown that in areas of high and stable transmission, malaria is the major contributor to anaemia which is responsible for more deaths than cerebral malaria [10-13]. A randomized controlled trial carried out in Tanzania showed that in highly endemic areas, malaria accounts for about 60% of anaemic episodes whilst iron deficiency contributes only 30%[11]. Another study in the same country which investigated several risk factors for anaemia including sickle cell status and nutritional status among others found that the single most important factor was *Plasmodium falciparum* parasitaemia [OR 4.3, 95% CI 2.9-6.5, P<0.001][14]. Malaria control with treated nets and chemoprophylaxis has also been found to reduce the level of anaemia (Elizeus). Studies in Northern Ghana found a significant drop in mean Hb levels of children under 5 years of age by the end of the high transmission season(Koram season) with no difference in anthropometric indices(Koram sev an)

1.2 Improving access to effective treatment for malaria

Much current research concentrates on preventing individuals (especially children and pregnant women) from becoming infected with malaria. There are currently a number of proven effective preventive measures including the use of treated nets and intermittent preventive treatment. Preventing disease is clearly the ideal, however, in areas of high endemicity, some infections will

occur even when good control measures are in place. Most deaths from malaria are preventable using currently available treatment, if it is applied early enough in the infection. Thus research on how to ensure that those who become infected do not go on to develop significant morbidity and mortality is important. Barriers to effective treatment may occur at many levels, ranging from failure by parents to recognize illness in a child until it is too late, difficulties in accessing health services leading to delayed presentation to inadequacy of diagnosis or treatment once medical services are accessed. These issues can be divided into problems in accessing appropriate health care and problems once health care is accessed (e.g problems with diagnosis or drug resistance). Many deaths are likely to be due to several factors combining to cause delay in receiving adequate treatment rather than to one single reason.

Some of the issues that influence access to and quality of treatment are considered below

Distance and travel costs

Distance from the health facility and travel costs have been identified by several authors as having significant effects on access to health care. They argue that these are more important than the actual cost of the services. In a Ghanaian study, the direct effect of user fees on utilization was found to be less significant than the effects of distance and travel costs [15]. In Tanzania and Guinea, mothers were more likely to use a public health facility if it was close by [16,17]. However, in Zaire, it was found that although travel distance had a significant negative effect on user's ability to choose their health facility it was less important than the actual price users paid for services [18,19]. Not all patients used the closest source of care in Thailand [20]. A study in Ghanaian rural communities discovered that children with severe illness were being treated at home because the parents could not afford the bills at the health facility only five kilometres away [21].

Perceived quality of service

Variables affecting quality of care include the number and types of health personnel available, the variety and consistency of drug supply and the physical condition of the facilities themselves [19]. In addition, provider attitude and behaviour reflect the quality of care from the user's perspective. Some authors believe that the cost of health services does not affect utilization as much as the quality of care. Results from some studies suggest that some of the harmful effects of user fees on access to care can be mitigated if fee income is used effectively to improve quality of care as perceived by the population [15,22-24]. A "natural experiment" in Cameroon that studied the effect of the introduction of user fees alongside improvements in the quality of services, found that proportionately more poor people sought care at facilities that also improved quality. Conversely,

the poorest were least likely to use the "control" health centres where no improvements in quality were achieved [24].

Socio-cultural factors

The experience of illness including its prevalence in the community, the frequency of infection of household members, the past history of episodes of illness in the affected individual, familiarity with and access to different treatment modalities and therapies and a variety of other social, factors are known to influence care and treatment [25-27]. In a Ghanaian study, belief in supernatural cause of illness led to delays in treatment because time was spent finding out what the cause of the illness was [21]. Previous interaction with the health system encourages use of the system for an illness episode [28]. The educational level of the mothers has a major influence on health related behaviour [29]. Rural-urban differences in health-seeking behaviour have also been observed [16].

Availability of services

The availability of services as well as the distribution and organization of alternative sources of care also affect the choice of sources of health care that people make. In some rural areas in Ghana, the only contact the inhabitants have with orthodox medicine is through untrained, semi-literate drug peddlers who move from one village to another to sell drugs and who sometimes give injections [21].

Cost of services

There is an on-going debate about the impact of user fees on utilization of and access to health services, especially by the poor [30].

Payment of (increased) health care fees has been shown to become an unacceptable burden on some households, leading to delayed or adapted treatment-seeking, use of informal and less effective sources of health care, and further impoverishment of marginalized families [21,31-33]Studies in Ghana, Zaire, Mozambique and elsewhere showed that the introduction of user fees was associated with a drop in utilization. In Ghana, when user fees were introduced in 1985, attendance dropped to a quarter of the 1984 levels with a gradual rise to the original level in urban areas but with persistence of low levels of attendance in rural areas [34]. A similar situation was observed in Mozambique [35]. In Zaire, an increase in the price of health care led to sharp falls in the demand for curative contacts and under-five clinic visits [36]. Bitran also showed in Zaire that moderate fees had a statistically significant negative effect on usage rates [18]. In Lesotho, the 0-5 year age group appeared to have been most affected [37]. Birch in his study of the recent experience of health service costs in the UK found that the non-exempt were 40% less likely to receive treatment than the exempt[38].

Examining the demand for outpatient services in one region of the Philippines, Akin concluded that price is not nearly as important a determinant of demand as usually assumed. They found that price increase in one source had actually caused some switch in favour of alternatives [22]which may not always be desirable. This study, however, was unable to study the differences by income groups. Gertler et al did and found price elasticity to be much higher for lower income groups[39]. Shaw and Griffin believe that the major disadvantage of some of these studies is that there is very little information on the socio-economic characteristics of users, the quality of services and other confounding factors and therefore the drop in utilization patterns following the introduction of fees may be over-stated [19,40].

Creese however cites several recent papers from utilization reviews in both low income and industrialized countries which provide evidence that the use of health services by the poor is more affected by price increases than is use by the rich. He contends that none of the demand studies so far refer to actual field experiments in which user fees were introduced and their effects studied [41]. Birch concludes that findings from the US Rand study imply that at least some of the service utilization deterred by charges leads to adverse health outcomes [38].

It is not, therefore, certain to what extent the financial barrier to health care is important in the control of diseases such as malaria particularly in children under five years. Various mechanisms aimed at addressing this have been tried in the past and are still being used.

1.3 Methods of overcoming financial barriers to obtaining effective treatment

Even the most optimistic studies of willingness and ability to pay find that some proportion of the population truly cannot pay and require assistance [19]. To avoid the problems associated with trying to identify those who cannot pay, some governments and ministries of health have instituted universal coverage with free primary care for identified groups such as children under five who are vulnerable to many infectious diseases including malaria. It is expected that reducing financial barriers will lead to prompt care for acute episodes of illness avoiding a chronic course that could result in severe disease such as severe malarial anaemia.

Stierle et al classified the mechanisms used for overcoming financial barriers into three main categories: differentiation of prices, subsidizing specific services and activities that address specific target groups (targeted subsidy) and exemption from payment [30]. Others have also instituted pre-payment schemes.

Price differentiation

Price differentiation which introduces lower prices for the target group than for all others may be based on socio-economic factors such as incomes, demographic factors such as age and gender or health factors such as infection with a contagious disease or a combination of these factors. Sometimes, in areas where a large proportion of the population is poor, prices are maintained at a low level throughout that community but set higher in other areas with higher purchasing power [30].

Subsidizing specific services and activities that address specific target groups

Subsidies of services or specific activities are recommended for activities that yield a high level of externalities e.g immunization. Subsidies help to deliver these services free or at a very low price to the poor who are usually the ones most in need of the services. Subsidies of specific activities are however not necessarily targeted to the very poor [30].

Exemption schemes

Stierle and others identify two types of exemption schemes: The first type links eligibility to professional status e.g civil servants whilst the other links eligibility to socio-economic or health status e.g poverty or age. Most countries implement either one or both types of the scheme in some form.

Pre-payment schemes

The major objective of such schemes is to protect subscribers from unaffordable health care costs at times when they are least able to pay, by collecting a contribution when their cash incomes are highest [42]. It is suggested that systems of pre-payment and health insurance may overcome some difficulties in financing health services and have the potential to facilitate access to health care. Insurance-like systems have an important advantage of separation of time of payment from time of use of services so that payment does not occur at the moment of greatest vulnerability. These systems do not directly solve the problem of permanent exclusion of the poor and vulnerable from health care but it may be possible to provide exemptions from premium payment for the very poor or vulnerable by having a third party such as local governments or other public institution, pay or subsidize their premium[30].

The Ghanaian Parliament by an Act last year established the National Health Insurance scheme, however, the only subsidies listed in the act relate to indigents. It has also been documented that the exemptions scheme for children under five is currently not effective.

It is not easy to set up intervention studies that investigate new approaches to payment for health care. Several studies in this area have, therefore, made use of time series data collected before and after the introduction of an intervention, or used multivariate statistical analyses of cross-sectional household data. Others have simulated user responses to surrogate measures of the intervention being studied [19]. Some studies have examined the impact of different kinds of financing schemes and levels of financial access on equity, utilization, efficiency of services, and other econometric indices. The limitations of these studies, which are unavoidable and accepted by the investigators, is that it is difficult to avoid the biases associated with differences in socio-economic status between the different groups being compared.

Randomized controlled trials are designed to eliminate bias, making comparison of two groups much easier in terms of the issue being assessed. Randomization, when applied to a sufficiently large number of individuals, will ensure that the different groups are similar at baseline. Thus any difference observed between groups is very unlikely to be due to measurement error or confounding, increasing the credibility of the inference that any difference observed is due to the intervention [43]. Though randomized trials have been carried out in the past in this area, such studies have looked at utilization of services as the main outcome. What is new about this trial is that it will be looking at objective measures of health outcomes in addition to utilization. The unit of randomization used by previous studies is groups of people who have chosen to access a particular health financing system. In this case, though the unit of randomization is the household, the impact of the randomly allocated intervention on the individual child less than five years in a particular household can be assessed.

We have therefore designed a randomized, controlled trial to determine the impact of improved financial access to health care on morbidity due to severe malaria and health service utilization in a rural area of Ghana. Malaria is the most commonly reported illness in this deprived rural area which is similar to most hyper endemic rural areas in Sub Saharan Africa. If we can show that reducing the financial barrier to care in a vulnerable group such as children less than five years of age leads to a significant reduction in malaria-associated endpoints (especially anaemia) this would provide strong evidence of the importance of financial access to treatment in preventing morbidity from malaria that would be of relevance to other malaria endemic areas in Africa.

2.0 RATIONALE OF THE STUDY

Malaria is a major cause of childhood morbidity and mortality through several mechanisms, especially anaemia and cerebral malaria contributing nearly 25% of child mortality in Africa. Effective control strategies depend both on preventing disease and on treating those who become infected. There are many barriers preventing access to prompt and effective treatment, some of which are amenable to intervention, others (such as distance to healthcare) less so. This study will concentrate on modifiable financial barriers to achieving effective treatment for malaria.

The study aims to determine by means of a randomized trial the impact of reducing financial barriers to health care on morbidity due to severe malaria among children 6 to fifty-nine months and on outpatient utilization. An existing pre-payment scheme in the study area will be utilized to improve financial access for half of 2168 households with eligible children who have not been enrolled.

The impact on severe anaemia, mean haemoglobin and anthropometric indicators will be assessed. Health service utilization will be measured in both groups by passive surveillance. Patients' perceptions of malaria and health financing will be explored. The study will contribute to the current debate on the relative importance of cost of care as a barrier to health care and provide evidence of the potential for the removal of this barrier as a strategy for malaria control, as well as methods to optimize this.

3.0 OBJECTIVES

To assess by means of a randomized trial, the impact of improving financial access to health care on morbidity due to severe malaria and utilization of health services among children 6 to 59 months of age at the end of the malaria transmission season..

1.3.1 Primary objective

To compare the prevalence of anaemia (Hb<8g/dl) among children six to fifty-nine months of age, from households randomized to improved financial access or without for one year.

1.3.2 Secondary objectives

1. To compare the proportions of children six to fifty-nine months of age with severe anaemia (Hb<6 g/dl) from households with or without improved financial access.

2. To compare the health service utilization rate among children six to fifty-nine months of age from households with or without improved financial access.
3. To compare the mean haemoglobin concentration among children six months to fifty-nine months of age from households with or without improved financial access.
4. To compare anthropometric indicators among children six to fifty-nine months of age from households with or without improved financial access.
5. To compare child (6 to 59 months) as well as household all cause mortality among households with or without improved financial access.
6. To examine primary and major secondary outcomes by socio-economic status of households.
7. To compare primary and major secondary outcomes among children from self-enrolled and trial-enrolled households.
8. To document community knowledge, attitude and practice with regards to severe malaria, user fees, exemptions, the existing pre-payment scheme and indicators of poverty.

4.0 METHODS

4.1 Study Design

The study is a two-arm open randomized controlled trial. It has a quantitative element based on pre-determined outcomes as well as a qualitative element.

4.2 Study Site

The study will be carried out in the Dangme West District in southern Ghana, a purely rural district with an estimated 2004 mid year population of 115,005. The population lives in scattered small communities of less than 2000 people. The main local language is Dangme. The district is divided into four administrative sub-districts. There are 4 health centres and 6 community based clinics in the district. Private sector facilities include two private clinics and one private maternity home. Currently, the doctor: population ratio is 1: 57,500 whilst the nurse: population ratio is 1:1,355. There are 300 traditional healers, a total of 92 trained traditional birth attendants (TBAs) & "wanzams" (local circumcisers) and an equal number of untrained TBAs who provide alternative medical services. There is one publicly owned laboratory based in the health centre situated in the district capital and two privately owned laboratories in two other sub-districts. The district currently has no hospital though one of the health centres is in the process of being upgraded to a district hospital. The inhabitants use all five surrounding hospitals for referral care as well as for some primary care.

Figure 2: Map of the Dangme West District showing its location in Ghana

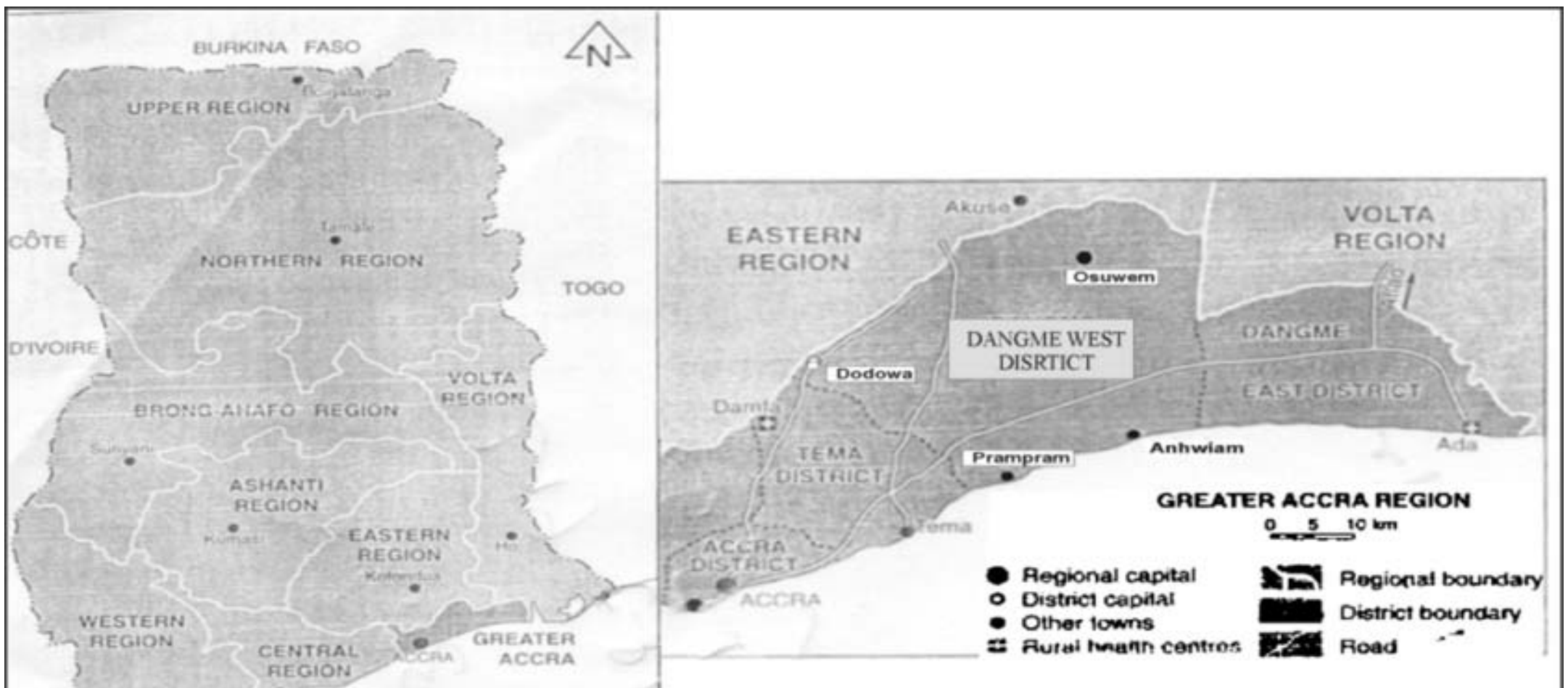

Dangme West District and its location within Ghana

Malaria control in the area is based on prompt diagnosis and presumptive treatment of cases with chloroquine. The use of insecticide treated nets is being promoted all over the country including this area and as such some subsidized nets have been sold in the past to pregnant women and children less than five years. A voucher system for pregnant women is planned. Malaria accounts for over 50% of reported cases at the outpatients department. There are no reliable recent estimates of infant or childhood mortality for the area. There are two main seasons; a rainy season from May to October and a dry season from November to April. Malaria transmission is highest during the rainy season. The district is made up of two different ecological zones: a coastal savannah and a forest zone. The coastal savanna area is mesoendemic whilst the forest area is hyperendemic for malaria. Home visit data suggest that the yearly incidence rate of malaria in the hyperendemic area is fairly constant at approximately 106.6 cases per 1000 population compared to 59 cases per 1000 population in the mesoendemic area. The average man-biting rate in the former area is 0.79 bites per man per night with parasite rates of 61.3% during the dry season and 68.3% during the wet season among children 2-9 years of age [44].

The Pre-Payment Scheme

The cost of treating a child with an acute episode of malaria in the publicly owned primary care health facilities in the district is approximately C10,000 (£0.75). Though exemption for children under five years became policy in the country in 1997, a recent review of the policy showed that only 6% of all children less than five years visiting public health facilities in Greater Accra Region where the study district is located were in practice exempted from paying fees for curative care [45].

As a step towards improving financial access to health care, the district initiated a pre-payment scheme which is currently in its fourth year of operation. Enrolment is by household. Registered members of the scheme, who pay C25,000 (£1.65) per adult and C15,000 (£1.00) per child or elderly, are allowed to use any of the ten primary care clinics in the district whenever they are ill without having to pay at the point of use, either for diagnosis or drugs. There is no limit to primary care usage by any member. In addition members of the scheme can be referred to any of five surrounding referral hospitals of their choice when indicated and given free treatment up to a ceiling of C400,000 (£25), beyond which they have to pay themselves.

Each member of an enrolled household receives a photo-ID card and has a unique identification number. Enrolled members who are ill are only required to present their ID cards at primary health care facilities in the district to receive free health services. Households not enrolled in the scheme, pay for their services out of pocket. Health facilities providing services to members of

the scheme send monthly returns covering the members they have seen during the periods and the drugs and services they were given. The primary care providers are paid for all services provided without limit. Referral hospitals also send similar returns on each patient seen and are either paid the total amount for the patient if the bill was less than C400,000 or the ceiling if it exceeded that amount. As agreed, the member would have been asked by the hospital to pay any excess over this ceiling.

The cost of drugs and services for the year are agreed upon at annual primary provider and referral hospital meetings. The study is using this existing scheme as a means of improving financial access to health care to the intervention group.

Data from the first year of operation of the scheme (2000/2001)(figure 1) compares utilization rates in primary facilities among the enrolled and non-enrolled.

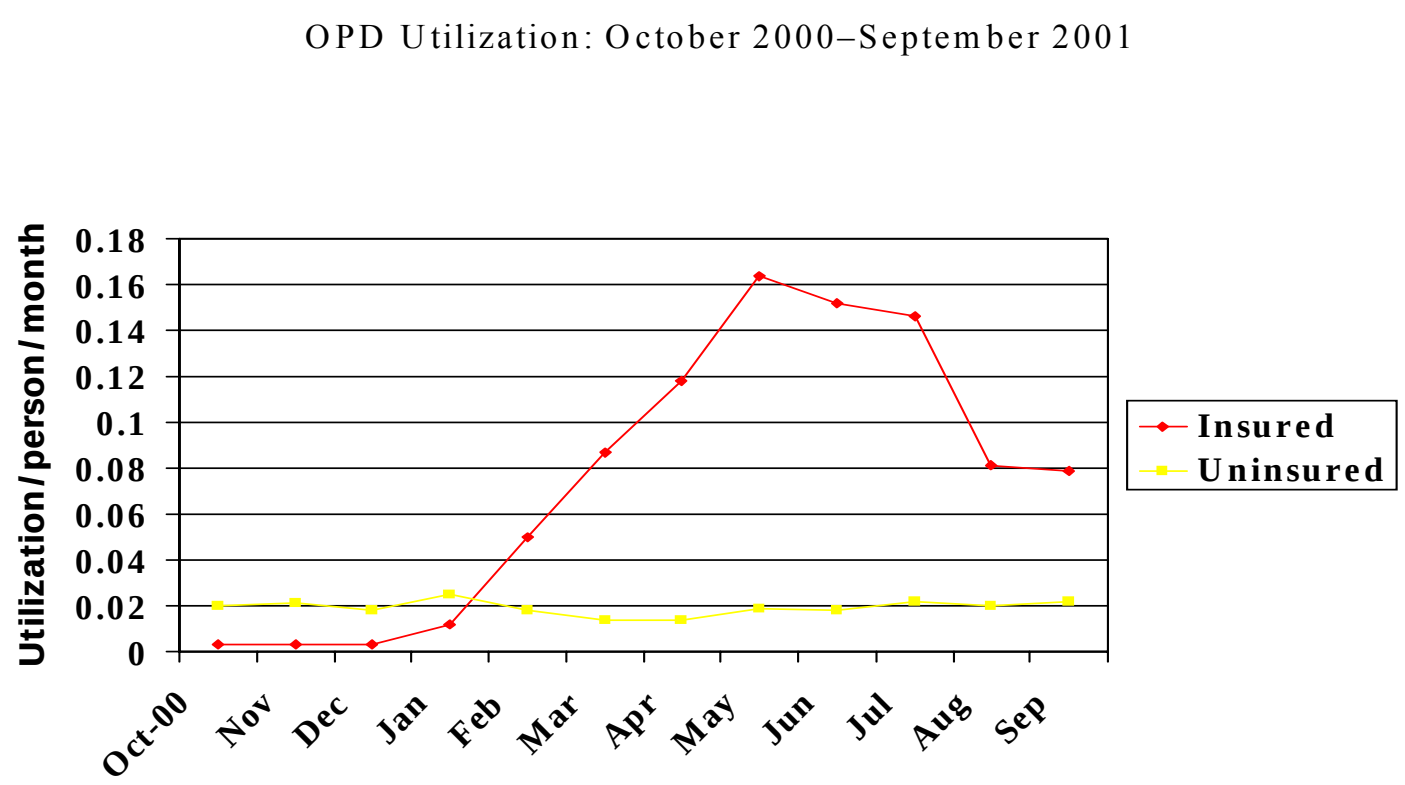

4.3 Study Population

All households in the Dodowa and Prampram sub-districts with at least one child 6 to 59 months who have not already enrolled in the pre-payment scheme for the year are eligible to participate in the study. This information was obtained from a data base of households at the District Health Directorate. There are approximately 11,622 households in the two sub districts and out of these, an estimated 2470 households have eligible children. 135 of these have enrolled voluntarily in the scheme.

2194 households with 2592 children will be randomly selected from among the households who have not enrolled for participation in the study. All the 135 enrolled households will be invited to participate as a subsidiary arm.

4.4 Inclusion and exclusion criteria

- Inclusion Criteria
  - Age documented to be between 6 and 59 months (verified by means of road to health charts or birth certificates).
  - Parental consent obtained.
  - Parents or guardians intend to remain in the study area for the next two years.
  - Household not already enrolled in pre-payment scheme.
- Exclusion Criteria
  - Parental consent refused.
  - Household due to emigrate from study area within two years.
  - Household currently enrolled.

4.5 Sample size

The primary end point for the trial will be the proportion of children with severe anaemia (Hb<6g/dl) in intervention and control groups at the end of the malaria transmission season. The prevalence of severe anaemia among children 6-24 months of age in Northern Ghana was found to be 22.1% at the end of the malaria transmission season [12]. The prevalence in Dangme West in Southern Ghana is likely to be lower. Therefore, we assume that the prevalence of severe anaemia among the control group will be 10% at the end of the 24-week period of follow-up. In order to detect an absolute difference of 4% in the prevalence of severe anaemia between the two groups, the sample size required which will give the study a power of 90% at a significance level of 5%, will be a total of 2028 children with 1014 in each arm. This sample size should also be able to detect a 0.3 g/dl difference in mean haemoglobin between the two groups.

4.6 Screening and enrolment procedure

Initial visits will be paid to all randomly selected households to explain the rationale for the study, and to seek their consent for participation. Consenting heads of households will then be invited to relevant community ballot meetings depending on the distance of their residence from the nearest health facility. Those agreeing to participate will provide written consent.

4.7 Randomization and allocation of intervention

A stratified randomization procedure will be used [46]. Households will be divided into three strata based on distance of residence from a health facility being ≤5km, 5-10km, and >10km

respectively since distance from a health facility is known to be a major determinant of its use. The aim is to address confounding that distance would introduce.

At the meeting all heads of households or their representatives will be allocated serial numbers. An equal number of folded papers with “Yes” or “No” written on them totalling the number present will be dropped into a rotating barrel and mixed up thoroughly in the view of all. Each household head will be invited to pick the papers by calling out their numbers. Those who pick “Yes” will be assigned to the intervention group and those who pick “No” to control. This process will be used to make the trial more acceptable to community members by showing them the lack of favouritism and randomness of the allocation [46]. Households will not be able to change their group until the study ends in December.

4.8 Initial cross-sectional survey

Visits to all households enrolled in the study will be carried out in May prior to the peak malaria transmission season. Characteristics of each household, including known risk factors for malaria such as type of housing, use of insecticide treated materials, use of other preventive measures for malaria and some indicators of household wealth will be documented. Each study child will be examined and a brief medical history obtained. The temperature of each child will also be recorded.

Similar data will be collected from eligible children of the households who voluntarily enroll in the pre-payment scheme in order to document the differences between them and the study participants in terms of health service utilization, health status, household wealth and other variables as a subsidiary part of the study

Anthropometric Measurements

All children will be weighed naked using infant weighing scales for children unable to stand alone and a mechanical stand-on scale for those who can stand alone. The recumbent length of children <24 months will be recorded using an infantometer and a stadiometer for the standing height of children >24 months of age. The mid upper arm circumference (MUAC) of each child will be measured using shakir’s strip. Measurements will be carried out twice and the mean used for analysis. In case of discrepancies a third measure will be carried out and the outlier discarded. Measuring devices will be calibrated daily. Anthropometric indices (body mass index, weight for age, weight for height, height for age etc) will be computed.

Haematological Measurements

A finger-prick sample of blood will be obtained for haemoglobin concentration determination using a Haemocue® microcuvette and a Haemocue haemoglobinometer.

Parasitological Measurements

Thick and thin blood films will be obtained from the same finger prick sample for parasitological measurements. They will be labeled with a unique study number for the index child, the community and date. The same study number will also be recorded on the child's record form. Both films will then be air dried and packed in slide boxes. The slides will be stained with Giemsa and examined by two independent microscopists at the Noguchi Memorial Institute for Medical Research. One hundred optical fields under 1000x oil immersion magnification will be examined before a film is recorded as negative. Counts of parasites in the thick film will be based on the number of asexual parasites per 200 white blood cells (WBCs). This number will be converted to parasites per microlitre of whole blood using a conversion multiple of 40 (assumes 8,000 WBCs per microlitre).

The presence of parasitaemia will be rapidly assessed in the field by means of a dipstick antigen capture test for all children with fever or reported fever in the past week. Children found to have haemoglobin concentration less than 8g/dl, fever or a history of fever and parasitaemia would be treated according to local guidelines and retained in the study.

4.9 Passive surveillance for malaria

Morbidity monitoring will be carried out using a combination of household and facility level passive case detection system. This will end in November. Mothers/Principal caretakers will be trained and provided with picture log sheets for each child 6 to 59 months to enable them to indicate both illness episodes and sources of health care for each month. In addition, particulars of all study children reporting at any of the ten primary care clinics as well as one private clinic in the area will be documented by health workers in notebooks prepared for that purpose.

For each child the health workers will document the name, study ID of child, date of visit, the diagnosis, as well as the outcome of the visit. All study participants who visit any of the primary care facilities will be treated by the health workers in accordance with the current standard treatment guidelines except in the case of uncomplicated malaria where a combination of amodiaquine and artesunate will be used instead of chloroquine, the current reccommendation. This is the proposed replacement for chloroquine as first line anti-malarial in Ghana.

Once a month, research assistants will visit referral hospitals to collect data similar to that being collected at the primary care facilities. In addition, data on referral and admission will be collected.

In the case of mortality, at a culturally appropriate time, a trained field worker will visit the home of the deceased child and a standard post-mortem questionnaire administered to the child's mother or caretaker. The results will be reviewed by three independent physicians to arrive at a possible diagnosis. If two out of three agree on a diagnosis of malaria, this diagnosis will be accepted. For children who visited hospital or died in the hospital, hospital case notes will be reviewed and where necessary, the attending physician will be interviewed for more complete information on the child's illness.

4.10 Final cross-sectional survey

A second, cross-sectional, household survey will be undertaken at the end of the peak malaria transmission season in November during which interviews, haematological, parasitological and anthropometric assessments relating to the children will be repeated. Data relating to the household will however not be collected in this round. Children found to have severe anaemia during the second survey will have a haematological work-up comprising Hb electrophoresis, full blood count, Glucose-6-phosphate dehydrogenase screen and stool examination.

4.11 Preliminary Assessment of Therapeutic Efficacy of Chloroquine

An assessment of the therapeutic efficacy of chloroquine will be carried out prior to the main study in order to ascertain the efficacy of chloroquine in the study area since the use of an ineffective drug will confound the impact of the intervention on the primary outcome, the haemoglobin concentration. Previous studies carried out in other sites in the country have revealed that chloroquine is no longer efficacious.

4.12 Background descriptive studies

Six communities will be randomly selected from communities less than 5km, 5 to 10km and greater than 10 km respectively away from the health facilities in the two sub districts. Data collection will involve mainly qualitative methods.

Focus group discussions will be conducted with 6 men and women groups separately. Each group will be comprised of between 8-12 discussants. Individual in depth interviews will be held with 2 traditional healers, 2 Traditional Birth Attendants, 5 community leaders and 2 owners of chemical shops. Four heads of facilities (which serve the communities in which the focus group discussions

and interviews will be conducted) will be interviewed. Additionally, a total of twelve care takers whose children have been admitted to the hospital with severe malaria will be interviewed.

4.13 Measurement of study end points

Primary

- Prevalence of anaemia (Hb<8g/dl) at the end of the malaria transmission season in each group

Secondary

- Prevalence of severe anaemia (Hb<6g/dl) at the end of the malaria transmission season in each group
- Mean haemoglobin concentration at the end of the malaria transmission season in each group
- Change in mean haemoglobin concentration in each group over the malaria transmission season
- Mean number of admissions to hospital with malaria per child in each group
- Mean number of outpatient visits to primary care and referral health facilities per child in each group
- Change in mean anthropometric indicators in each group
- Proportion of parasitaemic children in each group from both the baseline and final cross-sectional surveys
- All cause mortality among children in each group

4.14 Training for field work

The training of field staff will take about ten days. Trainees will be taken through

- The objectives of the study
- Community entry skills
- Collection of good quality data
- Interviewing techniques
- Laboratory and anthropometric measurement techniques

Questionnaires will be translated into the local languages and back into English to ensure that the understanding of the questions is the same among all interviewers. The tools will be pre-tested on two occasions and in two areas different from the area where the actual study will take place. After pre-test, the data collection tools will be finalized and an instruction manual produced for

interviewers. The best twelve among the trainees for laboratory and anthropometry will be selected and given more detailed training in laboratory and anthropometry techniques related to taking a good finger-prick sample, measuring haemoglobin using a haemocue machine, making of thick and thin blood films and collecting good quality blood samples for other haematological assessments. They will focus on this during the cross-sectional surveys.

5.0 DATA MANAGEMENT AND ANALYSIS

Field data will be collected by means of standard pre-coded questionnaires administered to household heads and mothers/principal caretakers of the children. *Q*uantitative data will be recorded on pre-coded questionnaires and record forms for anthropometric and haematological data whilst qualitative data will be recorded both on a tape recorder and in small notebooks. Data will be checked daily for accuracy and consistency, correctly labeled, and stored for data entry. For quantitative data, data entry clerks will carry out double entry of the raw data from questionnaires and record forms into Epinfo version 6. After data entry, data cleaning will be carried out to identify errors and inconsistencies for correction. Qualitative data will be analyzed using Textbase Alpha after converting the transcribed text file into an ASCII file. Standard notebooks will be used to collect data on all study participants who report at the clinic. Preliminary analysis of quantitative data will be carried out using Stata version 8.

For more detailed analysis at the end of the trial, an analytical plan will be produced. This will define the primary outcome measures including hospital admissions with malaria and malarial anaemia and describe the comparisons that will be made between the two study groups on one hand as well as between them and the group that enrolled in the scheme of their own accord on the other hand. Summary statistics, odds ratios, confidence limits and p-values will all be calculated to compare outcomes between the two groups for the primary and secondary endpoints. Stratified analysis will be carried out based on age, sex, distance from health facility, and household wealth. Means of continuous variables such as rise in haemoglobin count will be compared using the Student t-test. Geometric means will be used to compare parasite densities.

6.0 LIMITATIONS OF THE STUDY

There are a number of limitations of the study which are not very different from what pertains in other such studies but which have been taken into consideration and are being minimized as much as possible.

- There is a lower limit of quality of care beyond which giving free care will not make any impact. Care will be taken at all times to ensure that the quality of care in the health facilities is maintained in areas such as drug availability, staff availability among others.

- There is a potential limitation that can be caused by children in the study arm that has been enrolled being discriminated against because they are not paying for health care up front. This will be minimized if re-imbursements to health facilities are timely and staff bonuses instituted by the scheme for taking care of its members are paid regularly.

- The data relating to visits of the study children to the primary and referral facilities may not be completely captured by the health staff. This will however be minimized because the study children carry laminated ID cards which they have been advised to present each time they visit the clinic to enable staff record their details in the books provided. In addition, the clinic's own general consulting room registers are available to cross-check for the records of any child that might have been missed.

- Anaemia among children is known to be caused by several other conditions including helminthiasis and poor nutrition. However, in malaria endemic areas, malaria is known to be a major contributor to anaemia especially among children. This is especially important during the peak malaria transmission season. Randomization will also ensure that the contribution of these factors is the same in both groups especially since they appear very similar at baseline. Other causes of anaemia will be sought.

7.0 ETHICAL ISSUES

7.1 Ethical Approval

Ethical approval will be obtained from the Ethics committees of both the Ministry of Health/Ghana Health Service in Ghana and the London School of Hygiene and Tropical Medicine.

7.2 Ethical Considerations

Laboratory results will be communicated to the parents/guardians of the children concerned. All children who have clinical malaria, severe anaemia or severe malaria during the two cross-sectional surveys will be treated immediately at no cost in accordance with the Standard Treatment Guidelines of Ghana. All households agreeing to take part will get one year of pre-

payment made; the intervention group in year 1, and the control group in year 2. Additionally, those who have already elected to enrol will have a year paid for in year 2 to prevent any feeling of unfairness in the community. It will be made clear at the outset that the one year of payment is one-off, and will not be repeated.

Households will be given the option to withdraw at any time during the study with the assurance that this will not affect their normal care and management at the health facility nor their enrolment status. The results of the study will be communicated to the communities in which the data was collected.

7.3 Informed Consent

The PI will obtain written informed consent from parents or guardians of all study participants before their inclusion in the study. The rationale for the study, procedures, anticipated benefits and potential risks of the trial will be explained to them in their preferred language. Permission will be obtained from the chiefs and elders as well as other local authorities.

7.4 Confidentiality

All records of study participants will be kept in a manner that will conceal their identity but will be available for inspection by the supervisory panel and the District Health Directorate. Study records will be identified using study numbers of the participating children.

8.0 ADMINISTRATIVE MATTERS

8.1 Responsibilities of the Principal Investigator

The PI will be responsible for:

1. Recruiting and training all members of the investigation team.
2. Attending to all administrative and technical aspects of the study and ensuring smooth running of the trial, including adherence to the study protocol. She will also maintain a record of all correspondence concerning the study and a record of normal reference ranges for all laboratory tests.
3. Ensuring that all laboratory specimens are properly analysed.
4. Ensuring proper data collection and for carrying out data management and analysis.
5. Maintaining an inventory and use of patient records, study log books, drug supplies and dispensing, laboratory and anthropometric equipment and consumables as well as other study materials

8.2 Protocol Amendments

After the protocol has been finalized, no changes will be permitted without the agreement of the supervisor or the supervisory panel. Any change made will be formally endorsed and attached to the protocol.

9.0 TIME LINES AND MILESTONES FOR ACHIEVING OBJECTIVES

Activity	Year 2003				Year 2004				Year 2005			
	QI	Q2	Q3	Q4	Q1	Q2	Q3	Q4	Q1	Q2	Q3	Q4
Initial Preparations			■									
Assessment of therapeutic efficacy of chloroquine				■								
Community interviews				■	■							
Baseline cross-sectional survey						■						
Randomization & enrolment of households						■						
Passive surveillance						■	■	■				
Final cross-sectional survey								■	■			
Enrolment of households not enrolled in the first year									■			
Data Analysis and Report Writing									■	■	■	■

Q 1 – First Quarter; Q2 – Second Quarter; Q3 – Third Quarter; Q4 – Fourth Quarter

13.0 REFERENCES

1. Marsh K, Forster D, Waruiru C, Mwangi I, Winstanley M, Marsh V, Newton C, Winstanley P, Warn P, Peshu N ,et al. Indicators of life-threatening malaria in African children. *N Engl J Med* 1995:**332**:1399-404.
2. WHO. Twentieth Report. Expert Committee on malaria. 2000, World Health Organization.
3. MOH. Roll Back Malaria Strategic Plan for Ghana. 2000, Ministry of Health, Ghana.
4. GHAT. A quantitative method of assessing the health impact of different diseases in less developed countries. Ghana Health Assessment Project Team. *Int J Epidemiol* 1981:**10**:73-80.
5. WHO. Severe Falciparum Malaria. *Trans R Soc Trop Med Hyg* 2000:**94**.
6. Murphy SC ,Breman JG. Gaps in the childhood malaria burden in Africa: cerebral malaria, neurological sequelae, anemia, respiratory distress, hypoglycemia, and complications of pregnancy. *Am J Trop Med Hyg* 2001:**64**:57-67.
7. Menendez C, Fleming AF ,Alonso PL. Malaria-related anaemia. *Parasitol Today* 2000:**16**:469-76.
8. Fleming AF ,Werblinska B. Anaemia in childhood in the guinea savanna of Nigeria. *Ann Trop Paediatr* 1982:**2**:161-73.
9. Akenzua GI, Ihongbe JC, Imasuen IW ,Nwobi BC. Anaemia in children: a survey in (Obadan) a rural community in the rain forest zone of Nigeria. *J Trop Pediatr* 1985:**31**:20-4.
10. Snow RW, Bastos de Azevedo I, Lowe BS, Kabiru EW, Nevill CG, Mwankusye S, Kassiga G, Marsh K ,Teuscher T. Severe childhood malaria in two areas of markedly different falciparum transmission in east Africa. *Acta Trop* 1994:**57**:289-300.
11. Menendez C, Kahigwa E, Hirt R, Vounatsou P, Aponte JJ, Font F, Acosta CJ, Schellenberg DM, Galindo CM, Kimario J, Urassa H, Brabin B, Smith TA, Kitua AY, Tanner M ,Alonso PL. Randomised placebo-controlled trial of iron supplementation and malaria chemoprophylaxis for prevention of severe anaemia and malaria in Tanzanian infants. *Lancet* 1997:**350**:844-50.
12. Koram KA, Owusu-Agyei S, Utz G, Binka FN, Baird JK, Hoffman SL ,Nkrumah FK. Severe anemia in young children after high and low malaria transmission seasons in the Kassena-Nankana district of northern Ghana. *Am J Trop Med Hyg* 2000:**62**:670-4.
13. Stoltzfus RJ, Chwaya HM, Montresor A, Albonico M, Savioli L ,Tielsch JM. Malaria, hookworms and recent fever are related to anemia and iron status indicators in 0- to 5-y old Zanzibari children and these relationships change with age. *J Nutr* 2000:**130**:1724-33.
14. Kahigwa E, Schellenberg D, Sanz S, Aponte JJ, Wigayi J, Mshinda H, Alonso P ,Menendez C. Risk factors for presentation to hospital with severe anaemia in Tanzanian children: a case-control study. *Trop Med Int Health* 2002:**7**:823-30.
15. Lavy V ,Germain J. Tradeoffs in Cost, Quality and Accessibility in Utilization of Health Facilities: Insights from Ghana. In: Shaw RP, Ainsworth M (eds). Financing Health Services through User Fees and Insurance: Case Studies from Sub Saharan Africa. 1995, The World Bank: Washigton DC.

16. Glik DC, Ward WB, Gordon A ,Haba F. Malaria treatment practices among mothers in Guinea. *J Health Soc Behav* 1989:**30**:421-35.
17. Mtango FD, Neuvians D, Broome CV, Hightower AW ,Pio A. Risk factors for deaths in children under 5 years old in Bagamoyo district, Tanzania. *Trop Med Parasitol* 1992:**43**:229-33.
18. Bitran, Ricardo ,S B. Provider Incentives and Productive Efficiency in Government Health Services. 1992, Abt Associates: Bethesda MD.
19. Shaw P ,Griffin C, *Financing Health Care in Sub Saharan Africa through User Fees and Insurance*. First ed. Directions in Development. 1995, Washington DC.
20. Kaewsonthi S ,Harding AG. Cost and performance of malaria surveillance: the patients' perspectives. *Southeast Asian J Trop Med Public Health* 1986:**17**:406-12.
21. Asenso-Okyere WK, Anum A, Osei-Akoto I ,Adukonu A. Cost recovery in Ghana: are there any changes in health care seeking behaviour? *Health Policy Plan* 1998:**13**:181-8.
22. Akin JS, Griffin CC, Guilkey DK ,Popkin BM. The demand for adult outpatient services in the Bicol region of the Philippines. *Soc Sci Med* 1986:**22**:321-8.
23. Lavy V ,Quigley J. The Willingness to pay for the Equality and Intensity of Medical Care. 1993, The World Bank: Washington DC.
24. Litvack JI ,Bodart C. User fees plus quality equals improved access to health care: results of a field experiment in Cameroon. *Soc Sci Med* 1993:**37**:369-83.
25. Fitzpatrick R, Hinton J, Newman S, Scambler G ,Thompson J. The Experience of Illness. 1984: Tavistock.
26. Csete J. Health-seeking behavior of Rwandan women. *Soc Sci Med* 1993:**37**:1285-92.
27. Agyepong IA ,Manderson L. The diagnosis and management of fever at household level in the Greater Accra Region, Ghana. *Acta Trop* 1994:**58**:317-30.
28. Belcher DW, Nicholas DD ,Blumenfeld SN. Factors influencing utilization of a malaria prophylaxis programme in Ghana. *Soc Sci Med* 1975:**9**:241-8.
29. McCombie SC. Treatment seeking for malaria: a review of recent research. *Soc Sci Med* 1996:**43**:933-45.
30. Stierle F, Kaddar M, Tchicaya A ,Schmidt-Ehry B. Indigence and access to health care in sub-Saharan Africa. *Int J Health Plann Manage* 1999:**14**:81-105.
31. Gilson L. Government Health Charges: Is Equity being abandoned? *EPC Publication, London School of Hygiene and Tropical Medicine* 1988:**15**.
32. Russell S. Ability to pay for health care: concepts and evidence. *Health Policy Plan* 1996:**11**:219-37.
33. Mbugua JK, Bloom GH ,Segall MM. Impact of user charges on vulnerable groups: the case of Kibwezi in rural Kenya. *Soc Sci Med* 1995:**41**:829-35.
34. Waddington C ,Enyimayew K. " A Price to Pay, Part I: The Impact of User Charges in Ashanti-Akim District, Ghana". *Int J Health Plann Manage* 1989:**4**:17-47.
35. World B, *Better Health in Africa: Experience and Lessons Learned*. 1994, Washington DC.
36. DeBethune X, S A ,P L. The Influence of an Abrupt Price Increase on Health Service Utilization: Evidence from Zaire. *Health Policy Plan* 1989:**4**:76-81.

37. Byrne D ,Gertler P. An Analysis of the Impact of User Fees on Outpatient Utilization in Lesotho. 1990, The Rand Corporation: California.
38. Birch S. Health care charges: lessons from the U.K. *Health Policy* 1989:**13**:145-57.
39. Gertler P, Locay L, Warren C ,Sanderson. "Are User Fees Regressive?" *J Econometrics* 1987:**36**:67-88.
40. Yoder RA. Are people willing and able to pay for health services? *Soc Sci Med* 1989:**29**:35-42.
41. Creese AL. User charges for health care: a review of recent experience. *Health Policy Plan* 1991:**6**:309-19.
42. Lambo E. Aims and Performance of Pre-payment Schemes In: Sustainable Health Care Financing in Southern Africa. 1996, Economic Development Institute Health Policy Seminar: Southern Africa. p. 50.
43. Kirkwood BR, Cousens SN, Victora CG ,de Zoysa I. Issues in the design and interpretation of studies to evaluate the impact of community-based interventions. *Trop Med Int Health* 1997:**2**:1022-9.
44. Afari EA, Appawu M, Dunyo S, Baffoe-Wilmot A ,Nkrumah FK. Malaria infection, morbidity and transmission in two ecological zones Southern Ghana. *Afr J Health Sci* 1995:**2**:312-315.
45. Garshong B, Ansah E, Huijts I, Dakpallah G ,Adjei S. "We are still paying" A Study on the factors affecting the Implementation of the Exemptions Policy in Ghana. 2001, Ministry of Health / DANIDA.
46. Smith P ,Morrow R, *Field Trials of Health Interventions in Developing Countries: A Toolbox*. Second ed. 1996, London: Macmillan Education Ltd.

14.0 APPENDICES

Appendix 1 Information and consent form

INFORMATION AND CONSENT FORM

A randomized controlled trial of the impact of improved financial access to health care on morbidity due to severe malaria and health care utilization among children 6 months to 5 years of age in a malaria endemic area in Ghana

Field Worker's code ☐☐ **Date** ☐☐/☐☐/☐☐

1. Randomization Group ***(Indicate A, B or C as appropriate)*** ☐

2 Community [ ] **3.Community Code** ☐☐☐

4. Compound No ☐☐/☐☐☐☐

5. Mother's Name [ ]

6. Name of Household Head [ ]

7. Eligible Children

Name	ID No	Sex {}		Yes (1) No (2)
1	☐/☐☐/☐☐☐☐	☐	☐	☐
2	☐/☐☐/☐☐☐☐	☐	☐	☐
3	☐/☐☐/☐☐☐☐	☐	☐	☐
4	☐/☐☐/☐☐☐☐	☐	☐	☐
5	☐/☐☐/☐☐☐☐	☐	☐	☐

Information for Parents and Guardians

Every year about one million around the world including this district die from severe malaria (asraku). One of the reasons for these deaths is because their parents are unable to seek care promptly for them since they cannot afford it at the time of illness.

We plan to carry out a study to find out the impact of ensuring that every child under five years has access to health care at the time they are ill without thinking of payment. To do this, we have to screen and select about 2500 children, half of whom will be given a card that ensures free care for them under the *Dangme Hewami-nami kpee* scheme this year . The other half will also receive the same benefit next year though they will have to pay for health services as usual this year. This allocation is random and your child may fall into either group. This means that each participating household will be enrolled for one year only. We hope that you will be able to take part in the study.

During the study, tiny drops of blood will be taken on two occasions at home and any time your child visits the health facility from a finger-prick. This is to assess your child's "blood level" and to check for the presence of malaria parasites. Other measurements that include your child's weight, height and the thickness of his/her arm will be taken. In the process of collecting the blood sample, from the finger tip your child may experience mild discomfort. This does not represent a health risk to your child. None of these tests will cost you any money and in the process, you will find out about the health status of your child.

All the information collected about you and your child will be kept confidential and will only be made available to relevant authorities for the purpose of making important decisions and conclusions for this study.
You are at liberty to refuse or withdraw your child from the study at any time and this will not prejudice the standard of medical care your child is supposed to receive at the health facility. Any time after this child has been enrolled you are at liberty also to contact Dr. Evelyn Ansah / her assistant if you feel that you have not been adequately informed about the procedure, potential risks, benefits, rights as parents of this child, and indeed any other aspects of this study.

The contact address for Dr. Evelyn Ansah is as follows:

District Health Administration/Research Center
Dodowa

Consent Form

I have been adequately informed of the purpose of this study, procedures, potential risks, benefits and consequences.
I also understand that half of the children will receive the benefit of being enrolled in a scheme that will give them free care this year. I understand that I am at liberty to withdraw consent for my child's participation any time in the course of the study. I understand that the information obtained as a result of my child's participation will be treated as confidential and used only by the investigators, the LSHTM as well as the Ghanaian authorities.
The investigators have answered all my concerns. I agree to take part in this study.

Parent/Guardian Name Date □□/□□/□□

Witness:

I have adequately informed the parents or legal guardians of the child/ren the purpose of this study, its procedures, risks, benefits and consequences. I have answered the parents questions regarding the study conduct and explained their concerns about the child's participation in the study. I will be available to continue doing so in the course of the study if the need arise

Dr. Evelyn Ansah: Date □□/□□/□□

Appendix 2 Data collection tools

ANTHROPOMETRIC AND HAEMATOLOGICAL FIELD ASSESSMENT (AH) FORM

Field Worker's code ☐☐ **Date of examination** ☐☐/☐☐/☐☐

Community…………………………………………………………………………….

Time Examination begun ☐☐☐☐**am/pm**

1. House ID ☐☐ /☐☐/☐☐☐☐

2. Household ID ☐/☐☐☐☐

3. Name of Household head [ ]

4. Mother's Name [ ]

5. Child's Study ID ☐/☐☐☐☐/☐

6. Name of Child [ ]

7. Sex of child Male (1) Female (2) ☐

**8. Age (*in completed months. FW to check study ID)***	☐☐ Months
**9. Mid Upper Arm Circumference**	☐☐.☐**cm**
**10. Height/length**	☐☐☐.☐
**11. Weight**	☐☐.☐**kg**
**12. History of fever in the past week?**<br>Yes (1) No (2)	☐
**13. Temperature today(*FW to measure temperature and record)***	☐☐.☐**°C**
**14. Smear taken?**<br>Yes (1) No (*state why in space for comments*)(2)	☐
**15. Haemoglobin count**	☐☐.☐**g/dl**
**16.Dipstick Results**<br>Positive (1) Negative (2)<br>Not Applicable(no history of fever & child's temp below 37.5°C (99)	☐☐
**17. Body Mass Index (BMI = Wght/Height$^{2.}$** (*FW to leave this blank*)	☐☐**kg/m^2**
**Time Examination ended** ☐☐☐☐**am/pm**	

**18. Comments**

*The **difference** between the two results for **height/length should not exceed 5mm.** If it does two fieldworkers should retake measurements

Checked: Supervisor Code ☐☐ **Date**_____/____/04

HOUSEHOLD CROSS-SECTIONAL SURVEY (XS) QUESTIONNAIRE- CHILD DATA

Every year several in this district die from severe disease. As discussed with you earlier, we are carrying out a study to look at factors contributing to the development of severe disease and death among children under five years of age. We would therefore like to ask you a few questions concerning your household and your child/ren. We assure you that any information collected will be kept confidential. General findings will be made available to relevant authorities for the purpose of making important decisions and conclusions from this study.

***(*Interview to be conducted with household head and mother/principal female caretaker of child)**

Field Worker code ☐☐ **Date of Interview** ☐☐/☐☐/☐☐

Community…………………………………………………………………………
Time Interview begun ☐☐.☐☐ **am/pm**

A. ***Child Background Information***

1. House ID ☐☐/☐☐/☐☐☐

2. Household ID ☐/☐☐☐☐

3. Name of Household head ☐

4. Child's Study ID ☐/☐☐☐☐/☐

5. Child's Name ☐

6. Sex Male (1) Female (2) ☐

7. Date of Birth (*FW to check Birth Certificate/RTH Card)* ☐☐/☐☐/☐☐
DD/ MM / YY

8. Age (i*n completed months)* ☐☐ Months

9. **Number of <u>other</u> siblings less than five years old** ☐

10. Relationship of main respondent to child

Mother (1) Father (2) Grandmother (3) Grandfather (4)
Other specify ………………(5) Aunt (6) Uncle (7) ☐

B. <Child> Morbidity

**11. In this past year, that is from Easter of last year to now, has <child> been admitted to a hospital and slept overnight?**<br>Yes (1) No (2)	☐
**12. How many times was <child> admitted for malaria within the last year?**	☐times

**13. When was the most recent admission?**<br><br>Yesterday (1)<br>Two to three days ago (2)<br>A week ago (3)<br>Two weeks ago (4)<br>A month ago (6)<br>More than a month ago (7)<br>More than a year ago (8)<br>Child has never been admitted before (9)	□
14. Now we want to talk about your child's most recent illness. When was the last time your <child> fell ill?<br>Yesterday (1)<br>Two to three days ago (2)<br>A week ago (3)<br>Two weeks ago (4)<br>A month ago (6)<br>More than a month ago (7)<br>More than a year ago (8)<br>Child has never been ill before (9)	□
15. When <child> was last ill, where did you seek care for <child>? *(if child was sent to health facility, go directly to Q18 and indicate "Not Applicable" for Q16 and Q17)*<br>Home (1)<br>Drug Peddlar (2)<br>Chemical seller (3)<br>Traditional Healer (4)<br>Other. Specify……………………………………. (5)<br>Clinic (6)<br>Hospital (7)<br>Not Applicable (99)	□□
16. There are many reasons why children are not sent to a health facility when they are sick. In this instance what was the special reason *(tick all the spontaneous responses after listening carefully to the mother's report)*?<br>Too expensive. Could not afford it (1)<br>Transport costs too high (2)<br>Difficult to obtain transport (3)<br>Staff there are not technically competent (4)<br>Other(specify)…………………………………….(5)<br>Staff there are not nice to patients (6)<br>Know how to treat the illness at home (7)<br>Prefer to use herbal drugs (8)<br>Chemical seller closer (10)<br>Traditional healer/TBA closer (11)<br>Not applicable. Child taken to health facility (99)	□□

17. You have said that_______ and _________ and ______ are the reasons why <child> was not sent the health facility. Of all the reasons you have given me which is the most important *(Still use the options in Q16)*	☐☐

C. *Household expenditure on health care for child*

18. Now let's discuss expenditure for this last illness. How much did you spend on:

Place	Activity	Amount Spent
	Direct health care costs<br><br>Transportation to and fro<br><br>Other costs(gifts, food)	…………………………..cedis<br><br>…………………………..cedis<br><br>…………………………..cedis
	Direct health care costs<br><br>Transportation to and fro<br><br>Other costs(gifts, food)	…………………………..cedis<br><br>…………………………..cedis<br><br>…………………………..cedis
	Direct health care costs<br><br>Transportation to and fro<br><br>Other costs(gifts, food)	…………………………..cedis<br><br>…………………………..cedis<br><br>…………………………..cedis

Total costs ……………………………..cedis<br>Not Applicable. (99)	☐☐
19. Was enough money available in the house to pay?<br>Yes (1) No (2) Not Applicable (99)	☐☐
20. If enough money was not available, how was money obtained to pay?<br>Sold agricultural produce (crops, animals etc) to pay (1)<br>Provided paid labour to pay (2)<br>Sold personal effects to pay (3)<br>Money contributed by relatives/friends (4)<br>Other specify……………………………………... (5)<br>Pawned personal effects to pay (6)<br>Went for a loan (7)<br>Not applicable. Money was available at home (99)	☐☐
21. Who in the family decides that a mother should take her child for consultation when child is ill?<br>Mother (1) Father (2) Both parents (3)<br>Grandmother (4) Other (Specify)……………………… (5)	☐

D. Malaria and preventive measures

22. Does this household use any preventive measures for malaria ("asra" / "atridii")?<br>Yes (1) No (2)	☐
23. How do you usually protect <child> against malaria? (*Interviewer to prompt if a preventive method is not mentioned unprompted)*<br>i)Treated bed nets/curtains Yes (1) No (2)<br>ii)Untreated bed nets Yes (1) No (2)<br>iii)Mosquito netting on windows/doors Yes (1) No (2)<br>iv)Mosquito spray/coils Yes (1) No (2)<br>v)Burning of local herbs/orange peels Yes (1) No (2)<br>vi) Other (specify)……………………………………………………	☐ ☐<br>☐<br>☐
24. Does this household own a bed net ("mudo") ?<br>Yes (1) No (2) Not Applicable (99)	☐<br>☐
25. How many nets does the household own? (*indicate 99 if household does not own a net)*	☐<br>☐
26. How many nights has <child> slept under net during the past one week? (*indicate "99" if household does not own a net and "00" if the child did not sleep under net))*	☐☐
27. Has the net <child> slept in ever been treated with a chemical to kill insects and mosquitoes? (*indicate 99 if household does not own a net and"77" if they own one but child did not sleep in it)*<br>Yes (1) No (2) Not Applicable (99)	☐☐

Time Interview ended ☐☐.☐☐ **am/pm**

HOUSEHOLD CROSS-SECTIONAL SURVEY (XS) QUESTIONNAIRE-HOUSEHOLD DATA

(Interview to be conducted with household head and mother/principal female caretaker of child)

Field Worker code ☐☐ Date of Interview ☐☐/☐☐/☐☐

DD/MM/YY

Community……………………………………………………………………………

Time Interview begun ☐☐.☐☐ am/pm

House ID ☐☐/☐☐/☐☐☐

Household ID ☐/☐☐☐☐

Name of Household Head

Name of Mother ☐

Child/ren Study IDs ☐

☐/☐☐☐☐/☐

☐/☐☐☐☐/☐

☐/☐☐☐☐/☐

A .Household Background Information

1. How many people are there in this household? (*Those who share economic resources or eat from the same pot. We will define these as your household members)*	☐☐
2. How many of your household members are less than five years of age?	☐
3. What is your main occupation<br>Farming (1) Fishing (2) Civil servant (3)<br>Artisan (4) Other. Specify……………………… (5)<br>Trader (6) Unemployed (7)	☐
4. What is your religion?<br>Christian (1) Muslim (2) African Traditional (3)<br>Agnostic (4) Other (specify)……………………....(5)	☐
5. What is your ethnic group?<br>Dangme (1) Ga (2) Akan (3)<br>Ewe (4) Other (specify)……………… (5)<br>Krobo (6) Northern/Upper (7)	☐
6. Marital status of main respondent Married (1) Single (2) Separated (3) Divorced (4) Widowed (5)	☐
7. Number of years of formal education completed by mother/principal caretaker	☐☐
8. Number of years of formal education completed by household head	☐☐

(*if different from mother/principal caretaker. Indicate '99' if same person is mother/principal caretaker)*	
B. Perception of Malaria	
9. What is the commonest illness that affects children in this area?<br>Malaria/ 'asra'/fever (1)<br>Convulsion (2)<br>Worms (3)<br>Diarrhoeal diseases (4)<br>Other (specify)…………………………………… (5)<br>Measles (6)<br>Stomach pain (7)	☐
10. What causes malaria ("asra")?<br>Mosquito bite (1)<br>Too much sun (2)<br>Worms (3)<br>Poor nutrition (4)<br>Other (specify)…..………………………………….. (5)<br>Eating unripe fruit (6)<br>Eating oily foods (7)<br>Don't know (88)	☐
11. Does malaria ("asra") ever become severe in children and kill them?<br>Commonly (1)<br>Sometimes (2)<br>Infrequently (3)<br>Never (4)	☐
C Health Facility Utilization	
12. Can you tell me which the closest health facility to your home is?<br>Dodowa health centre (1)<br>Prampram health centre (2)<br>Agomeda community clinic (3)<br>Doryumu community clinic (4)<br>Other specify…………………………………….. (5)<br>Kordiabe community clinic (6)<br>Dawhenya community clinic (7)<br>Ebenezer clinic (8)<br>Grace Maternity Home (9)	☐
13. From which health facility do you usually seek care for <child/ren>?<br>Same as above (1)<br>A hospital (2)<br>Traditional healer/TBA (3)<br>Other (specify)…………………………………... (5)<br>Chemical Seller (6)<br>Drug peddler (7)	☐

Treat all illnesses at home myself (8)
Child has never been ill (9)

14. How do you usually get there?
By private car (1)
By taxi (2)
By tro-tro (3)
By Motor-bike (4)
Other. Specify……………………………………… (5)
By bicycle (6)
By walking (7)
Not Applicable. (99)

15. How long does it take you and how much does it cost you to get there? Time (in minutes) ………………………………mins

Cost ………………………………cedis

D. Mortality

16. In this past year, that is from Easter of last year to now, how many children under five years in this household has died from a febrile illness?
None (1) One (2) Two (3)
Three (4) Other specify…………………… (5)

F. Pre-payment Scheme

17. Have you ever heard about the Dangme Hewaminami Kpee (health insurance scheme)?(*If NO go directly to Q23 and indicate "99" forQ17 to Q22)*
Yes (1) No (2)

18. How did you hear about it?
Through friends (1) Through Radio (2) leaflets/brochures (3)
Through health staff (4) Others (Specify)……………………… (5)
Information van (6) Gongong beating (7) Assembly men (8)
Not applicable (99)

`19. Have you ever enrolled in the scheme?
Yes (1) No (2) Not Applicable (99)

20. Have you enrolled this year?
Yes (1) No (2) Not Applicable (99)

21. Why did you not enroll this year?
No Money (1)
Poor quality drugs (2)
Poor quality of health care offered at the clinic (3)
Staff reception poor (4)
Other (specify)…………………………………………… (5)
I live too far away from the nearest clinic (6)
I did not hear when the registration was going on (7)
Don't understand it (8)

I think premiums are too high (9)<br>Prefer to take the risk of falling ill (10)<br>Had traveled during registration period (11)<br>Not Applicable (99)	
22. Why have you never enrolled?<br>No Money (1)<br>Poor quality drugs (2)<br>Poor quality of health care offered at the clinic (3)<br>Staff reception poor (4)<br>Other (specify)………………………………………… (5)<br>I live too far away from the nearest clinic (6)<br>I never hear when the registration is going on (7)<br>Don't understand it (8)<br>I think premiums are too high (9)<br>Prefer to take the risk of falling ill (10)<br>Not Applicable (99)	☐☐

G. Exemptions

23. Does the government provide free health services for any category of people?<br>Yes (1) No (2) Don't Know (88)	☐☐
24. Which category of people are entitled to these free services? (*Do not prompt. Allow respondent to list as many as possible)*<br>Children under five mentioned (1)<br>Children under five not mentioned (2)<br>Don't know (88)<br>Not Applicable (99)	☐☐
25. Do you know anybody who has benefited from this before?<br>Yes (1) No (2)<br>Not Applicable (99)	☐☐

H. Poverty

26. What indicates that a household in this area is poor? (*Multiple answers allowed. Prompt client with "Is that all?" Tick all responses given)*<br>The kind of clothing they wear (1)<br>The number and quality of meals they have in a day (2)<br>The kind of house they live in (3)<br>The assets they own (4)<br>Other. Specify…………………………………………… (5)	
27. How do poor households survive in this area? (*Multiple answers allowed. Prompt client with "Is that all?" Tick all responses given)*<br>Borrow from family members<br>(1)<br>Borrow from neigHbours and friends<br>(2)	

Helped by family members (3)<br>Helped by neigHbours and friends (4)<br>Other. Specify………………………………………… (5)<br>Provide labour for money (6)	

H. Household wealth

28. Who owns this house?<br>Household owns it (1)<br>Family house(jointly owned) (2)<br>Belongs to a relative. Not paying rent (3)<br>Renting it from someone (4)<br>Other. Specify……………………………….(5)<br>Provided by govt/employer (6)	☐
29. What is the main lighting fuel of this household?<br>Electricity (1)<br>Gas (2)<br>Kerosene (3)<br>Candles (4)<br>Other. Specify………………………………..(5)	☐
30. What type of fuel does your household mainly use for cooking?<br>Electricity (1)<br>LPG/ natural gas (2)<br>Kerosene (3)<br>Charcoal (4)<br>Other (specify)………………………………(5)<br>Firewood (6)<br>Dung (7)	☐
31. What is the source of drinking water for this household?<br>Piped water (into residence) (1)<br>Piped water (public tap/neigHbour's house) (2)<br>Well/Borehole (3)<br>River/stream/pond/lake (4)<br>Combination.specify………………………………..(5)<br>Rain water (6)<br>Tanker truck (7)<br>Pure water (8)	☐
32. Time to water source (*to the nearest 10 minutes)*<br>Less than 10 minutes (1)<br>10 minutes (2)<br>20 minutes (3)<br>30 minutes (4)<br>40 minutes (6)<br>50 minutes (7)<br>1 hour or more (8)	☐
33. What sort of sanitation facility does this household use?	

Flush toilet (used by household only) (1)<br>Flush toilet shared (2)<br>Own KVIP/ bucket/pan/ traditional pit latrine (Household) (3)<br>Shared KVIP/ bucket/pan/ traditional pit latrine (4)<br>Other specify……………………………………………...(5)<br>Public Toilet (6)<br>No facility/bush (8)	☐
34. What is the main floor material of the house (*Record observation)*<br>Ceramic tiles/terrazzo (1)<br>Cement (2)<br>Brick/cement blocks (3)<br>Mud/sand/earth (4)<br>Other……………………………………………..(5)<br>Zinc/Asbestos/Aluminum sheets (6)<br>Tarpaulin, plastic sheets (7)<br>Asbestos (8)	☐
35. What is the main material of the walls?(*Use options for Q34)*	☐
36. What is the main material of the roof? (*Use options for Q34)*	☐
37. How many households share this compound?<br>One (1)<br>Two (2)<br>Three (3)<br>Four (4)<br>Five (6)<br>Six (7)<br>More than six (8)	☐
38. How many rooms in this compound belong to this household?<br>One (1)<br>Two (2)<br>Three (3)<br>Four (4)<br>Five (6)<br>Six (7)<br>More than six (8)	☐
39. Does this household own:<br>A radio Yes (1) No (2)<br>A Television Yes (1) No (2)<br>A Refrigerator Yes (1) No (2)<br>A Telephone Yes (1) No (2)	☐<br>☐<br>☐<br>☐
40. Does any member of this household own:<br>A bicycle? Yes (1) No (2)<br>A motor-cycle? Yes (1) No (2)<br>A tractor? Yes (1) No (2)<br>A fishing vessel? Yes (1) No (2)<br>A commercial vehicle? Yes (1) No (2)	☐<br>☐<br>☐<br>☐<br>☐<br>☐
41. Does this household own:	☐

A subsistence farm? Yes (1) No (2)<br>A commercial farm? Yes (1) No (2)<br>Land Yes (1) No (2)<br>Cattle? Yes (1) No (2)<br>House/s for renting out? Yes (1) No (2)	☐<br>☐<br>☐<br>☐
42. How much average income per month has this household been getting over the past six months from the work you do? *(FW to get the total for 6 months and divide by 6)*<br>……………………………………cedis	
43. How much average monthly remittance have you received as remittance from relatives/friends over the period?<br>……………………………………cedis	
44. Have you had to borrow any money over the same period (six months)?<br>Yes (1) No (2)	☐
45. How much in total have you had to borrow over the period?<br>…………………..cedis Not Applicable (99)	☐☐

***Thank you very much for your very useful answers and your time*.**

Time Interview ended ☐☐☐☐am/pm

CARE PROVIDER KEY INFORMANTS INTERVIEW GUIDE

1. PERSONAL INFORMATION

Name	Occupation
Address	Area of responsibility
Age	Sex

2. INFORMATION ABOUT HEALTH AND MALARIA

- What are the most important disease conditions affecting people in this area?
- What are the most important conditions that lead to severe illness among children in this community?
- What types of febrile illnesses affect children in this community?
- What disease conditions lead to death among children in this community?
- What other sources of care are there for caretakers of children with these conditions?

3. INFORMATION ABOUT THE HEALTH FACILITY AND HEALTH CARE SEEKING BEHAVIOUR FOR MALARIA

- Where do you provide your services? Is there any place set aside for delivering services?
- What facilities are available for delivering the services?
- Where do people who seek care from you come from?
- What type of patients come to seek care from you?
- What sort of severe conditions do you see? (***If severe malaria not mentioned, probe for severe malaria)***
- Which type of patient normally presents to you with severe malaria?
- How many severe malaria episodes do you see per day or per month?

4. INFORMATION ON MALARIA AND ITS COMPLICATIONS

a)What signs and symptoms do children with severe malaria usually present to you with?
(If not mentioned, go on to ask specifically about the following complications of malaria)

- Anaemia
- Recurrent Convulsion
- Inability to sit or stand
- Unrousable coma/unconsciousness
- Failure to eat or drink
- Repeated vomiting
- Deep breathing
- Change in mental status

b) For each of the conditions above, ask for the following

- Local name
- Types
- Perception on cause
- Prevention and treatment.
- How long have patients usually had their symptoms prior to consulting you?
- Where do you think patients go first for treatment of the above symptoms?
- When they come to you how you do treat the children with these symptoms?
- What are the main reasons for delay in seeking treatment for these symptoms?

5. REFERRAL PRACTICES

- Do you refer cases?
- Who/ where do you refer to?
- What is the referral procedure?
- Under what circumstances do you refer children? Please illustrate with the last case you referred.
- Are there any delays in the referral procedures?
- Do you get feedback from the institutions or persons to whom you usually refer?

6. PAYMENT FOR SERVICES

- How do mothers whose children you look after pay for your services?
  (Probe for cash , kind, installment or all at once)
- Do you offer credit facilities?
- How much does it cost to treat a case of simple malaria?
- How much does it cost to treat a case of severe/complicated malaria?
- How do your charges compare with those of the government facilities?
- What happens if a mother cannot pay for services?
- How can you tell if a mother genuinely cannot pay for services?

7. OTHER KEY INFORMANTS

- Are there other local people you can suggest who would be useful to talk to?

Thank you very much for your time and for giving us insight into these important issues affecting health in this area.

INTERVIEW SCHEDULE: COMMUNITY KEY INFORMANTS

1. PERSONAL INFORMATION

Name	Address
Sex	Age
Occupation	Education
Number of children	Number under five

2. INFORMATION ABOUT THE KEY INFORMANT'S ROLE

What is their field of responsibility?

3. INFORMATION ABOUT HEALTH AND MALARIA

What is the most important disease leading to death in adults and in children in this community?
What signs and symptoms does the person associate with severe malaria? **(*Note all signs and symptoms mentioned)***

4. INFORMATION ABOUT HEALTH CARE

Where do mothers go for advice and treatment when their children are very sick?
Who in the family decides that the mother should take a sick child to hospital?

5. INFORMATION ABOUT SEVERE MALARIA

a) What types of febrile illnesses are there in this community? (If not mentioned, ask about malaria. Go on to ask specifically about the following complications of malaria

- Anaemia
- Recurrent Convulsion
- Inability to sit or stand
- Unrousable coma/unconsciousness
- Failure to eat or drink
- Repeated vomiting
- Deep breathing
- Change in mental status

b) For each of the conditions above, ask for the following

- Local name
- Types
- Perception on cause
- Signs and symptoms
- Prevention and treatment.
- Sources of care
- Constraints to seeking help
    - How long does it take to get to the nearest health center or hospital, during the rainy season and at other times?
    - What factors delay seeking care and treatment promptly?

6. PERCEPTIONS ON PAYING FOR HEALTH CARE

- How do you pay for bills when a child is sick
- Big bills Small Bills
- Ask for what the group means by either big or small bills

- If you did not use the money for health care, what would you have used it for?
- Have you heard of the DHK
    a. What have you heard about it
    b. From whom
    c. Are you members
    d. What are some of the benefits
- Perceptions and experience with the scheme
- Knowledge about government exemption policy
    e. Source of knowledge
    f. Who benefits
    g. Perceptions and experience with exemptions for children under 5

7 PERCEPTIONS ON POVERTY

1. How would you describe a poor person or household in this area
2. How would you describe a rich person in this area
3. How do poor people survive in this area
4. What are the existing community structures for helping poor people in this community

Thank you very much for your time and for giving us insight into these important issues affecting health in this area.

FOCUS GROUP DISCUSSION GUIDE FOR MOTHERS WITH CHILDREN LESS THAN FIVE.YEARS OF AGE

1. BACKGROUND INFORMATION

Name	Marital Status
Age	Educational Status
Sex	Number of children
Occupation	Number Under 5

2. GENERAL INFORMATION ON HEALTH AND TREATMENT SEEKING BEHAVIOUR

1. What disease conditions in this community lead to severe illness in children?
2. What disease conditions in this community lead to death in children?
3. What are the available sources of health care for mothers with sick children in this community?
4. At what stage of the illness do mothers take their children for consultation?
5. Who in the family decides that a mother should take her sick child for consultation?
6. Who gives advice to the mother/caretaker when a child is sick?
7. Does a woman need permission to send her child for health care?
8. Under what circumstance does this happen?
9. How long does it take to get to the nearest health facility?
10. What factors delay seeking health care and treatment promptly?

3. INFORMATION ON MALARIA AND ITS COMPLICATIONS

What types of febrile illnesses are there in this community? (If not mentioned, ask about malaria. Go on to ask specifically about the following complications of malaria

- Anaemia
- Recurrent Convulsion
- Inability to sit or stand
- Unrousable coma/unconsciousness
- Failure to eat or drink
- Repeated vomiting
- Deep breathing
- Change in mental status

For each of the conditions above, ask for the following

- Local name
- Types
- Perception on cause
- Signs and symptoms
- Prevention and treatment.

4. PERCEPTIONS ON PAYING FOR HEALTH CARE

2. How do you pay for bills when a child is sick
3. Big bills Small Bills
4. Ask for what the group means by either big or small bills
5. If you did not use the money for health care, what would you have used it for?
6. Have you heard of the DHK

    a. What have you heard about it
    b. From whom
    c. Are you members
    d. What are some of the benefits
7. Perceptions and experience with the scheme
8. Knowledge about government exemption policy
    a. Source of knowledge
    b. Who benefits
    c. Perceptions and experience with exemptions for children under 5

5. PERCEPTIONS ON POVERTY

1. How would you describe a poor person or household in this area
2. How would you describe a rich person in this area
3. How do poor people survive in this area
4. What are the existing community structures for helping poor people in this community

Thank you very much for your time and for giving us insight into these important issues affecting health in this area.
